# PacDOCK: A Web Server for Positional Distance-Based and Interaction-Based Analysis of Docking Results

**DOI:** 10.3390/molecules27206884

**Published:** 2022-10-14

**Authors:** Jacopo Carbone, Alessia Ghidini, Antonio Romano, Luca Gentilucci, Francesco Musiani

**Affiliations:** 1 Department of Chemistry “G. Ciamician”, University of Bologna, Via Selmi 2, 40126 Bologna, Italy; 2 Laboratory of Bioinorganic Chemistry, Department of Pharmacy and Biotechnology, University of Bologna, Viale G. Fanin 40, 40127 Bologna, Italy; 3 Wiraki, Via Laurentina 749, 00143 Rome, Italy

**Keywords:** web server, molecular docking, atom matching, RMSD calculation, protein–ligand interactions, clustering, molecular visualization, structure-based drug design, binding mode, docking assessment

## Abstract

Molecular docking is a key method for structure-based drug design used to predict the conformations assumed by small drug-like ligands when bound to their target. However, the evaluation of molecular docking studies can be hampered by the lack of a free and easy to use platform for the complete analysis of results obtained by the principal docking programs. To this aim, we developed PacDOCK, a freely available and user-friendly web server that comprises a collection of tools for positional distance-based and interaction-based analysis of docking results, which can be provided in several file formats. PacDOCK allows a complete analysis of molecular docking results through root mean square deviation (RMSD) calculation, molecular visualization, and cluster analysis of docked poses. The RMSD calculation compares docked structures with a reference structure, also when atoms are randomly labelled, and their conformational and positional differences can be visualised. In addition, it is possible to visualise a ligand into the target binding pocket and investigate the key receptor–ligand interactions. Moreover, PacDOCK enables the clustering of docking results by identifying a restrained number of clusters from many docked poses. We believe that PacDOCK will contribute to facilitating the analysis of docking results to improve the efficiency of computer-aided drug design.

## 1. Introduction

Molecular docking is one of the most employed techniques in modern drug design and structural biology [1,2,3,4]. Specifically, the goal of protein–ligand docking is to predict the pose(s) of a small molecule (*ligand*) into the binding site of a biological macromolecule, such as a protein (*receptor*) [5]. To perform a structure-based docking study, the 3D structure of the receptor is required, while the structure of the ligand can be created from scratch. In the last decade, the tremendous increase in the number of protein structures determined experimentally using X-ray crystallography, nuclear magnetic resonance (NMR) spectroscopy, or cryogenic electron microscopy (cryo-EM) has prompted a wide use of docking strategies [6,7,8]. The binding pose of the ligand is defined not only according to receptor and ligand shape but also depends on receptor–ligand intermolecular interactions, which altogether permit establishing a docking score, thereby approximating the binding affinity [9]. As mentioned, docking programs aim at predicting the optimal pose of the ligand in the binding pocket through the generation of several poses and conformations of the ligand on the receptor surface that are ranked according to the docking score. Two principal measurement types, positional distance-based and contact-based, can be used in order to compare and evaluate docking results [10]. Regarding positional distance-based measures, the most common is the root mean square deviation (RMSD) of Cartesian coordinates of the atomic positions. RMSD is a quantitative measure that represents the average deviation of a binding pose with respect to a reference orientation and conformation (which can be an experimental binding pose) in the 3D space. RMSD is calculated as follows:

(1) $$\mathrm{RMSD}=\sqrt{\frac{1}{N}\sum_{i=1}^{N}\delta_{i}^{2}}$$

where *N* is the number of atoms in the ligand and *δ_i_* is the Euclidean distance between the *i*th pair of corresponding atoms. Positional measurements such as the RMSD must be interpreted with caution and can be combined with measures based on interactions and visual inspection of docking results [11]. Among contact-based measures, the interaction-based measures consist of the investigation of the key interactions the ligand forms with its receptor. Interaction-based measures play a crucial role in the evaluation of docking results, but also in the case of the comparison with experimental results, where the presence of key interactions between the ligand and the receptor is ascertained [12,13]. No single measurement is robust alone; rather, the synergistic combination of positional distance-based and interaction-based measurements allows a more complete and accurate interpretation of results [10].

There is no systematic method to assess *a priori* the best docking algorithm and protocol to use for a specific system. Consequently, the usual practice is to validate and optimise the algorithm and protocol used through the reproduction of experimental structural data. “Re-docking” refers to the procedure of taking an experimental structure of a target complex, removing the ligand, and then re-docking the ligand into the target [5]. Thus, the evaluation of re-docking results allows benchmarking of docking algorithms and protocols for the system of interest [14]. Once the accuracy of the docking method and protocol has been assessed, they can be used for the prediction of binding poses regarding new potential ligands through in silico pre-screening campaigns.

The analysis of docking results can be challenging, as several docked poses are usually computed, and different binding poses with nearly equal scores may be determined. To simplify the analysis, it can be particularly convenient to cluster the results to determine the similarity between the predicted binding modes and also the most probable binding poses. Cluster analysis defines a set of techniques aimed at grouping data into categories, called clusters, on the basis of their similarities [15]. Clustering reduces the number of poses that need to be examined since further analyses can only consider a representative pose from each cluster [16]. To perform this, a matrix of pairwise RMSD values is calculated and docked poses are clustered according to an RMSD cut-off, hence based on their positional distance.

These standard evaluation methods are apparently trivial tasks, but they can pose some challenges [17]. First, the RMSD calculation requires a direct atomic correspondence between the reference structure and the docked poses, but this correspondence is not always guaranteed. Indeed, some of the most commonly used free—or freely available for non-commercial research—docking software (e.g., AutoDock 4 [18], AutoDock Vina [19], OpenEye FRED (SDF) [20], OpenEye HYBRID (SDF) [20]) may generate an output structure file where the ligand atoms are in a different order with respect to the input molecular structure file. Thus, a program that also performs atom matching is required for the correct calculation of the RMSD between different structures. In recent years, two web servers for the calculation of RMSD have been developed: DockRMSD [21] and LigRMSD [22]. However, the latter services are limited to the RMSD calculation, and each allows only one specific input format. Moreover, in the case of LigRMSD, the input must be provided in a format not widely used in recent times (a ZIP file containing the reference and the docked poses in MOL format), and DockRMSD does not provide a visualisation tool. For these reasons, a single program that can execute atom matching and RMSD calculation with input files in different chemical formats represents a significant improvement.

Additionally, since cluster analysis can be performed with several different methods, principal docking programs that execute clustering employ different algorithms. Hence, cluster analysis is strictly linked to the docking program used. Consequently, there arises a need for a versatile tool to perform cluster analysis of docking results from different docking programs, with different cluster algorithms and parameters chosen by the user.

Furthermore, all these tasks are usually executed independently using different software, which can render these analyses a tedious and time-consuming process.

Finally, visual inspection of the receptor–ligand interactions remains a mandatory step in the evaluation of a reliable binding pose [23]. Nevertheless, this complementary analysis is not offered in conjunction with the calculation of the RMSD by the web servers DockRMSD and LigRMSD.

As a result, all these non-trivial analyses are necessary for the assessment of docking simulations. Therefore, the evaluation of docking results has always been one of the most challenging problems in computer-aided drug design [24]. Although docking algorithms are evolving to answer increasingly refined and complex problems, such as taking into account the receptor’s flexibility, considering the presence of water molecules in the binding cavity, handling large molecule databases during a virtual screening campaign, or docking on nucleic acids [25,26,27], one aspect that requires attention remains the careful choice of the docking protocol to be used. Herein, we present PacDOCK, a freely available online platform where the analysis and the visualization of docking—particularly re-docking—results can be run on a single web server with great flexibility. To achieve this goal, we developed new tools for positional distance-based and interaction-based analysis of docking results. Moreover, PacDOCK is also easy to use for novice users, and it includes extensive tutorials and documentation, as well as suggestions on how to choose the calculation parameters and interpret the results.

## 2. Results

PacDOCK is currently composed of three separate tools: (*i*) ProRMSD, which employs graph theory to perform atom matching and calculate the RMSD between a reference structure and the calculated binding poses, using different chemical file formats; (*ii*) PacVIEW, which offers two dedicated visualisation modes for inspection and comparison of docked poses and receptor–ligand interactions; (*iii*) ClusDOCK, which offers three algorithms to perform cluster analysis of different docking programs outputs, with user-selected parameters.

### 2.1. ProRMSD

ProRMSD enables the calculation of the RMSD between a reference molecular structure (for instance, one that have been experimentally determined) and different poses of the same molecule, usually the result of a docking simulation.

*Inputs*. ProRMSD requires two different files as input: a first file containing one or more molecular structures (e.g., the file generated by a docking program with the predicted poses of the ligand), and a second file containing the same molecular structure in the reference binding pose (e.g., a specific binding mode or the ligand found in the X-ray crystal structure). Normally, different docking programs work with different chemical file formats; thus, several file manipulations may be required before RMSD calculation. One of the conveniences of using ProRMSD arises from the fact that input files can be submitted in different common chemical file formats, such as SDF, PDB, MOL2, and MOL. This is possible since files are handled by OpenBabel [28], which converts them into SDF format, read by ProRMSD. Hydrogen atoms are not considered in the RMSD calculation. To avoid issues in the format conversion, both input files should be written in the same format. Finally, but not less important, ProRMSD offers the possibility to simultaneously confront a reference pose with several different poses generated by the docking program.

**Figure 1:**
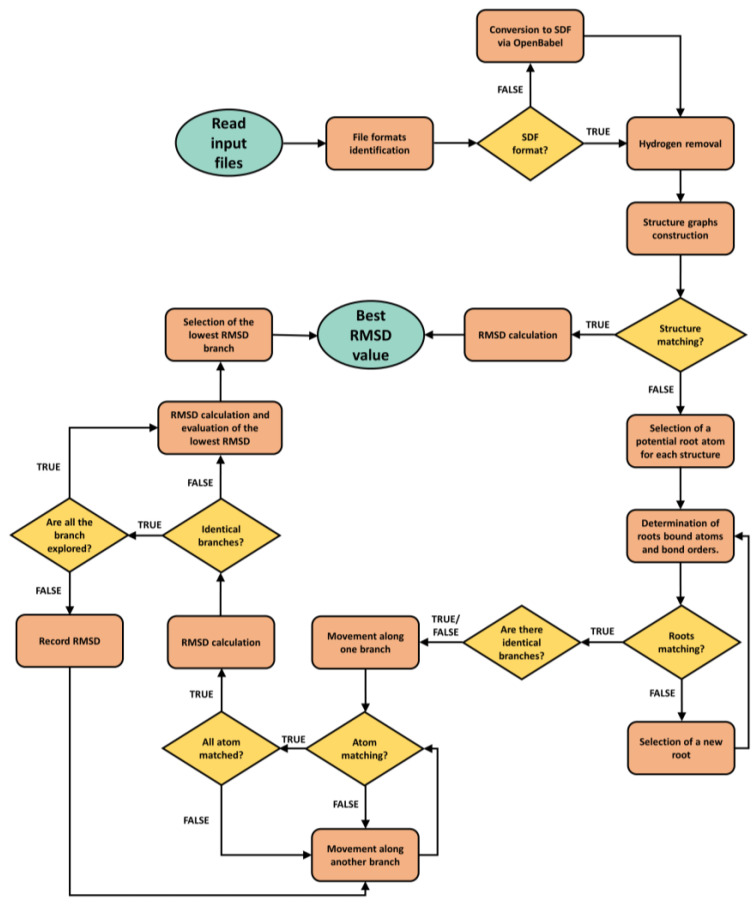
Flowchart of the ProRMSD algorithm.

*Processing method.* Since ProRMSD performs atom matching, it is also possible to calculate RMSD when atoms are randomly labelled. This has been achieved by representing every molecular structure as a graph, where the atoms are the nodes, and the bonds are the edges. Using the depth-first search (DFS) algorithm [29], the innovative idea is to identify the corresponding atoms of the two structures by following the same path from an atom selected as the root to the leaves, matching the same atoms in the two molecular structures between which the RMSD is calculated. The match between two atoms, one in the reference structure and the other in the predicted pose, is based on: (*i*) the atom type, (*ii*) the covalently bound atoms, and (*iii*) the order of such bonds. The matching is based on a greedy algorithm, which divides the problem into stages, aiming to find the optimal result at each stage using heuristics. Therefore, all possible paths are ranked at each branching step, and the branch that minimises the RMSD is followed. We opted for this strategy since the graph isomorphism problem is a non-polynomial problem [17]. In this class of problems, the computation time increases dramatically with the number of atoms in the considered molecule. Using the local approach, the computation time is reduced and it is also possible to calculate the RMSD with a good level of accuracy for molecules with a high number of atoms (see below and the Supplementary Data for some examples and comparison with similar web servers). The flowchart of the algorithm is reported in Figure 1.

*Output*. PacVIEW output consists of interactive 3D structures of loaded files. Each loaded structure can be displayed in multiple style modes (licorice, ball and stick, cartoon, surface, etc.), with colours selected by the user. In addition, the user can not only rotate and translate the structures, but also choose different parameters regarding the visualisation and save images at different resolutions. For more details, users could refer to the related documentation on the PacDOCK web server.

*Calculation modes.* ProRMSD can execute two different types of calculation: (*i*) “RMSD”, which calculates RMSD on all heavy atoms of the molecule, and (*ii*) “Backbone RMSD”, which is helpful in the case of peptide ligands and permits one to calculate RMSD only on the molecular backbone of a peptide, excluding the peptide side chains from the calculation. The server default mode is the RMSD calculation on all heavy atoms of the molecule.

*Output.* The output generated by ProRMSD is the numeric value of RMSD in angstroms (Å). Each predicted molecular structure returns its own value of RMSD when compared to the reference structure.

### 2.2. PacVIEW

PacVIEW enables 3D graphic visualisation to study and interpret docking results with a user-friendly interface. It offers two visualisation modes: (*i*) “Receptor–Ligand interactions” and (*ii*) “Docking results”.

*Inputs*. In the Receptor–Ligand interactions mode, PacVIEW requires two different files as input: a first file containing the target receptor and a second file containing its ligand (e.g., a docked pose or the ligand found in the X-ray crystal structure). In the Docking results mode, PacVIEW needs as input a file containing one or more molecular structures (i.e., the file generated by a docking program with the predicted poses of the ligand). A second file containing the same molecular structure in the reference binding pose (e.g., the ligand found in the X-ray crystal structure) can be loaded to study the differences between the poses. Input files can be submitted in different common chemical file formats, such as PDB, PDBQT, SDF, MOL2, MOL, ENT, CIF, and GZ.

*Visualisation modes*. The Receptor–Ligand interactions mode allows the visualisation of the ligand into the binding pocket of its receptor. The great advantage of this mode lies in the possibility of easily visualising the key interactions between the ligand and the receptor. Moreover, the user can choose the types of interactions to be investigated (e.g., hydrogen bond, ionic interaction, hydrophobic contact, π-stacking, metal coordination). The Docking results mode shows the pose(s) of a docking result, which can be compared with a reference structure. This can be useful to visualise the same input files used in the ProRMSD calculation to completely study the positional and conformational differences.

### 2.3. ClusDOCK

ClusDOCK enables the clustering of docking results by creating a pairwise RMSD matrix and by identifying a limited number of clusters from a large number of docked poses. ClusDOCK is useful for clustering binding poses in the case of software that do not include this possibility (e.g., OpenEye FRED or OpenEye HYBRID), or for re-clustering binding poses by giving the user a choice of both algorithm and clustering parameters, or for clustering binding poses obtained by merging several docking calculations performed with different random seeds. Exactly because docking scoring functions are imperfect, a clustering operation can be useful to identify binding poses that occur more often than others independently of the scoring function itself. This procedure is implemented by several docking programs, such as HADDOCK [30,31] or DOCK [32] for example.

*Input.* ClusDOCK requires a single file, which is the output of one of the following docking programs in their specific file format: AutoDock Vina (PDBQT format), OpenEye FRED (SDF), OpenEye HYBRID (SDF). However, outputs generated by additional docking programs may be used for clustering following future updates.

*Processing method*. The novelty of ClusDOCK lies in its versatility, which allows the users to choose both the clustering algorithm and the parameters used in the calculation, i.e., the clustering cut-off and the minimum cluster size. ClusDOCK can execute the cluster analysis with three different algorithms: the gromos algorithm [33] and two different agglomerative hierarchical algorithms, namely single-linkage and complete-linkage clustering [15]. At the basis of each method, there is the calculation of an *N* × *N* matrix in which each element contains the RMSD of the *i*th binding pose calculated with respect to the *j*th binding pose.

When using the gromos algorithm, the number of other structures for which the RMSD is under the cut-off (called *neighbours*) is calculated for each structure. The structure with the highest number of neighbours represents the centre of the most populated cluster and, together with all its neighbours, forms the first cluster. The structures of this cluster are thereafter eliminated from the pool of structures. The assignment of the cluster centre is repeated until the pool of structures is empty, creating all possible clusters. Instead, when using the agglomerative hierarchical algorithms, the structures or clusters of structures that have the lowest RMSD are merged at each stage if the RMSD value is lower than the cut-off. Differences between the methods arise because of the different ways of defining the RMSD between two clusters or a structure and a cluster.

In single-linkage clustering (or nearest-neighbour method), the RMSD between two clusters is equal to the lowest RMSD between two structures: one in one cluster and one in the other.

In complete-linkage clustering (or farthest-neighbour method), the RMSD between two clusters is equal to the highest RMSD between two structures: one in one cluster and one in the other.

None of the above methods can be arbitrarily recommended above all others, and it has to be recognised that different clustering methods may give different results on the same data. For this reason, it could be useful to run analyses with different choices to check for robustness. ClusDOCK uses the gromos algorithm as the default one, which enables the definition of a series of non-overlapping clusters of structures with an easy and fast procedure. A potential benefit of applying the single-linkage algorithm is that it can be used to identify outliers since these structures are left as singletons; thus, they are not included in any of the clusters. Complete-linkage is the opposite to single linkage and may generate clusters that include very different poses [15]. When using the gromos algorithm, the cut-off affects the number of neighbour conformations and, thus, the definition of clusters. When using agglomerative hierarchical algorithms, the cut-off defines when the algorithm stops and, thus, the “optimal” number of clusters. The ClusDOCK default cut-off is 1.0 Å. The minimum cluster size is the number of structures that a cluster must contain so that this group can be considered a cluster. The ClusDOCK default minimum size is 4 structures. The selected value should depend on the number of compared poses: if only a few poses are clustered, then this value should not be very high, but if the analysis concerns many poses, a higher value can be used to avoid creating too many clusters with too few elements.

*Outputs.* ClusDOCK generates two different outputs: a table and a bar plot. The table contains the number of conformations found in each the cluster and their specific indexes (referring to the input submitted by the user), where the first one is the most representative of the cluster, as well as its scoring function. The y-axis of the bar plot shows the number of conformations for each cluster, whilst the x-axis shows the scoring function (determined by the docking program) for each cluster. In the case of the gromos algorithm, the representative structure is the centroid of each cluster. However, in the case of the two hierarchical algorithms, for each cluster, the one with the lowest score is chosen as the representative structure.

### 2.4. Usage Examples

In this section, we discuss two examples of PacDOCK usage for the complete analysis of docking results with RMSD calculation, visualisation of the docked pose with respect to the reference pose and receptor–ligand interactions, and clustering. The purpose of this section is to show the potential of PacDOCK in some typical cases of re-docking, without intending to go into the merits of the individual scientific problem.

The first application case regards the structure of the transmembrane domain of human metabotropic glutamate receptor 5, a class C G-protein-coupled receptor (GPCR), in a complex with the negative allosteric modulator 3-chloro-4-fluoro-5-[6-(1H-pyrazol-1-yl)pyrimidin-4-yl]benzonitrile (PDB ID 5CGC) [34]. The ligand binds the receptor via a combination of interactions, including hydrogen bonds, π-stacking, polar, and hydrophobic interactions [34]. GPCRs are a vast superfamily of eukaryotic transmembrane receptors which act as ubiquitously expressed key regulatory elements. This application case was selected since GPCRs constitute more than 30% of current drug targets [35].

**Figure 2:**
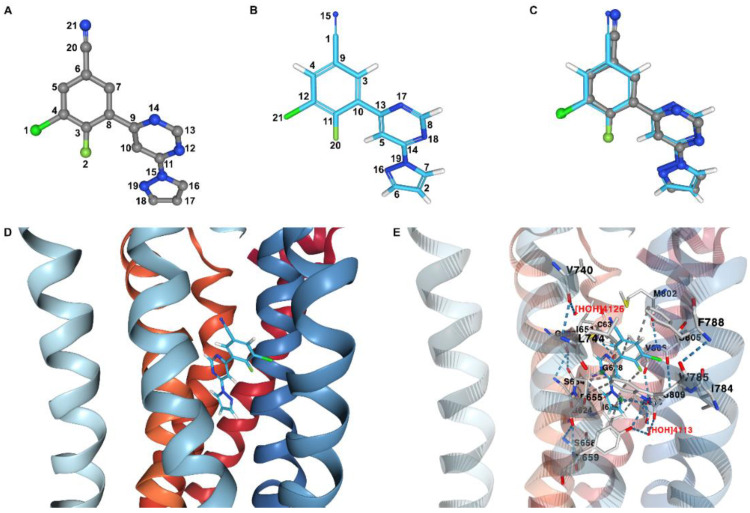
Analysis of re-docking results regarding a human class C GPCR receptor (mGlu5-StaR) with (3-chloro-4-fluoro-5-[6-(1H-pyrazol-1-yl)pyrimidin-4-yl]benzonitrile)—a negative allosteric modulator—bound in the allosteric site. Experimental pose of the ligand in ball and stick representation, with carbon, nitrogen, chlorine, and fluorine atoms coloured grey, blue, vivid green, and soft green, respectively. Docked pose of the ligand in liquorice representation, with carbon, nitrogen, chlorine, fluorine, and hydrogen atoms coloured light blue, blue, vivid green, soft green, and white, respectively. Receptor in cartoon representation, with atom index colour style. (**A**) Atom indexes of the experimental pose of the ligand. (**B**) Atom indexes of the docked pose of the ligand assigned by OpenEye HYBRID docking program. (**C**) Ligand docked pose superimposed onto the ligand experimental pose. (**D**) Ligand docked pose within the receptor allosteric site. (**E**) Interactions between the receptor and the ligand in the docked pose within the allosteric site. Selected residues from the receptor are in liquorice representation, with carbon, oxygen, nitrogen, and sulphur atoms coloured light grey, red, blue, and yellow, respectively. These residues are labelled in black, and selected interactions are shown as dashed lines. Water molecules are coloured and labelled in red. The ligand atoms involved in the main interactions are surrounded by a green sphere. The receptor cartoon representation has been opacified for clarity.

*ProRMSD*. Starting from the structural file downloaded from the RCSB Protein Data Bank [36], the ligand was removed from the structure and then re-docked into the target using the docking program OpenEye HYBRID (see Supplementary Data (S1) for details of the docking procedure). From the docking program output, the first pose of the ligand was used to create the input file for the RMSD calculation of this pose with respect to the experimental one in the protein–ligand complex, by using ProRMSD with the default RMSD calculation mode. The atom matching procedure is required for the RMSD calculation since the atoms in the experimental and the docked pose are labelled differently (Figure 2). Through the use of ProRMSD, it is also possible to calculate RMSD in this case, where atoms are randomly labelled. The atomic order in the structure files before and after the matching is shown in Supplementary Tables S1 and S2. The value of RMSD calculated between the reference and the docked pose is 0.297 Å (see Supplementary Table S3 for the RMSD calculation comparison with other web servers capable of performing similar calculations: DockRMSD [21] and LigRMSD [22]).

*PacVIEW*. The same two structures used for the RMSD calculation with ProRMSD were visualised using PacVIEW in the Docking results mode. As shown in Figure 2, it is possible to visualise the spatial differences between the two poses of the ligand. Moreover, the ligand was displayed within the allosteric site of the receptor, and the key interactions were investigated by using PacVIEW in the Receptor–Ligand interactions mode (Figure 2).

*ClusDOCK*. The first ten poses from the re-docking were clustered with ClusDOCK by using the gromos algorithm, with a cut-off of 0.5 Å and a minimum cluster size of 1 structure (the pairwise RMSD matrix is reported in Supplementary Table S4). The cluster analysis shows the grouping of the ten poses in four different clusters. Interestingly, the first cluster, and thus the most populated, is also the one with the most favourable scoring function (additional information on the clustering can be found in Supplementary Table S5, and the results are shown in Supplementary Figures S1–S3).

The second application case regards the molecular structure of the complex between a minor groove-binding drug (netropsin) and the DNA dodecamer d(CGCGATATCGCG), solved at a resolution of 2.4 Å (PDB ID: 1DNE) [37]. Netropsin is a DNA-binding compound that has a binding preference to stretches of AT-rich sequences. In the complex, netropsin binds to the central ATAT tetranucleotide segment in the narrow minor groove of the dodecamer B-DNA double helix through a combination of interactions, including hydrogen bonds, ionic charge attractions, and van der Waals interactions [37]. This application case was chosen since DNA–ligand docking is still a very challenging topic [38]. Indeed, nucleic acids differ considerably from proteins in their chemical and physical properties. For this reason, docking algorithms and scoring functions developed for protein–ligand interactions may fail for these receptors.

**Figure 3:**
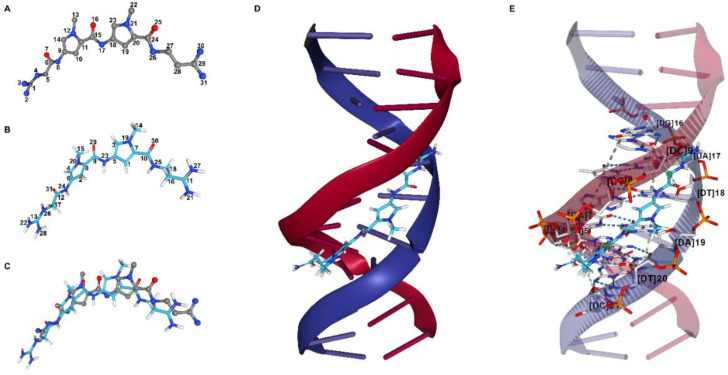
Analysis of re-docking results regarding the minor groove-binding drug netropsin and the DNA dodecamer d(CGCGATATCGCG). Experimental pose of the ligand in ball and stick representation, with carbon, nitrogen, and oxygen atoms coloured grey, blue, and red, respectively. Docked pose of the ligand in liquorice representation, with carbon, nitrogen, oxygen, and hydrogen atoms coloured light blue, blue, red, and white, respectively. DNA stands are in cartoon representation, while the nitrogenous bases are in base representation, with atom index colour style. (**A**) Atom indexes of the experimental pose of netropsin. (**B**) Atom indexes of the docked pose of netropsin assigned by OpenEye HYBRID docking program. (**C**) Netropsin docked pose compared with experimental pose. (**D**) Netropsin docked pose within the DNA minor groove. (**E**) Interactions between the DNA dodecamer and netropsin in the docked pose. Selected residues from the receptor are in liquorice representation, with carbon, oxygen, nitrogen, and phosphorus atoms coloured light grey, red, blue, and orange, respectively. These residues are labelled in black, and selected interactions are shown as dashed lines. Netropsin atoms involved in the main interactions are surrounded by a green sphere. The receptor cartoon representation has been opacified for clarity.

*ProRMSD*. The input file for the RMSD calculation of the first pose of netropsin re-docking was obtained, as reported in the previous example. The RMSD of this pose with respect to the experimental one was calculated by using ProRMSD with the default RMSD calculation mode. Using ProRMSD, it is also possible to calculate RMSD in this case, where the atom matching procedure is required since the atoms in the experimental and in the docked pose are labelled differently (Figure 3). The atomic order in the structure files before and after the matching is shown in Supplementary Tables S6 and S7. The value of RMSD calculated between the reference and the docked pose is 2.219 Å (see Supplementary Table S8 for the RMSD calculation comparison with DockRMSD [21] and LigRMSD [22]).

*PacVIEW*. The same two structures used for the RMSD calculation with ProRMSD were visualised using PacVIEW in the Docking results mode. As shown in Figure 3, it is possible to visualise the spatial differences between the two poses of the ligand. Moreover, the ligand was displayed within the DNA minor groove, and the key interactions were investigated by using PacVIEW in the Receptor–Ligand interactions mode (Figure 3).

*ClusDOCK*. The first twenty poses from netropsin re-docking were clustered with ClusDOCK by using the gromos algorithm, with a cut-off of 1.0 Å and a minimum cluster size of 1 structure (the pairwise RMSD matrix is reported in Supplementary Table S9). The cluster analysis shows the grouping of the twenty poses in seven different clusters. Interestingly, the first cluster, and thus the most populated, is also the one with the most favourable scoring function (additional information on the clustering can be found in Supplementary Tables S10–S14 and the results are shown in Supplementary Figures S4–S6).

An additional, exhaustive example of results obtained with PacDOCK is reported in the Supplementary Data. PacDOCK has been utilized for the complete analysis of docking results, RMSD calculation, visualisation of the docked pose with receptor–ligand interactions, and clustering of the agonist opioid peptide DAMGO in the μ-opioid receptor (μOR)-G_i_ protein complex (PDB ID: 6DDF) [39]. Molecular modelling in the opioid field is particularly relevant to rational drug design, since the different classes of ligands have distinct modes of receptor interaction and, for the agonists, activation [40].

## 3. Discussion

As the use of molecular docking methods grows, so does the need for programs that allow docking results to be completely analysed and easily interpreted. In this context, we developed PacDOCK, a free web server that aims to provide useful tools for a complete analysis of docking results through the comparison of molecular conformations and interactions, evaluation of docking methods, and molecular visualisation.

As shown in the usage examples section, ProRMSD proved to be an efficient tool that made it possible to calculate the RMSD even in the case of randomly labelled atoms, with a quick and easy calculation. The result obtained is comparable to other similar web servers, but what makes ProRMSD a good choice for RMSD calculation is the flexibility and ease of use of the program. In contrast to similar web servers, ProRMSD does not require conversions as it accepts multiple chemical file formats, permits one to compare multiple structures at the same time, and allows one to calculate different types of RMSD with a fast calculation (e.g., limiting the RMSD calculation to the backbone of a peptide ligand, a function not available in similar web servers). See Supplementary Tables S20–S32 and Supplementary Figures S11–S20 for a comparison with other web servers on ten re-docking calculations performed on a set of ligands chosen for their wide variety of molecular geometries and functional groups. When comparing ProRMSD with DockRMSD, differences were obtained in only a few cases. Even assuming that it is ProRMSD that gives the wrong result, our algorithm always leads to a very small error (not higher than an absolute difference of 0.028 Å and a relative difference of 1.55%). With respect to the comparisons between ProRMSD and LigRMSD, a higher number of differences can be found. However, in most of the cases, the values calculated with ProRMSD that differ from LigRMSD are instead in accordance with the values determined with DockRMSD. If we assume that both ProRMSD and DockRMSD are giving the wrong results, absolute differences of 0.50 Å are reached only in 2 cases among the 100 considered. On the other hand, manual atom matching and consequent calculation of the RMSD carried out for the first binding pose of each example showed that our algorithm is successful in the majority of cases. However, the error made remains nevertheless very small. Consequently, the use of a greedy algorithm was found to be efficient and appropriate for calculating RMSD in the case of protein–ligand docking, whilst also demonstrating considerable performance in terms of execution speed.

PacVIEW represents an important integrative tool which can be used after performing an RMSD calculation with ProRMSD. In this manner, the numerical value of RMSD between poses can be associated with a display of the spatial differences of the docking result with respect to the reference pose. Furthermore, PacVIEW can provide interaction-based insights that, combined with positional distance-based information, allows a complete and more reliable interpretation of docking results. PacVIEW has the benefit of being a free web viewer that does not require the installation of any packages on the user’s computer.

ClusDOCK emerges as a versatile tool, where the clustering algorithm and its parameters can be chosen. In addition to its flexibility, another advantage of ClusDOCK lies in the simplicity in which docking results can be analysed through the support of graphs and tables. It can be especially useful when several docked poses have similar scores, or a large number of docked poses have been computed. In this latter case, if numerous clusters are not generated, this can be considered an indication of poor convergence of the docking algorithm used, requiring cautious evaluation from the user.

Given the need to analyse docking results quickly and efficiently, PacDOCK represents a single online and free platform for a complete evaluation of docking results.

## 4. Materials and Methods

PacDOCK is a collection of tools running on the web. ProRMSD and ClusDOCK are written in Python 3.7 using OpenBabel 3.0.0 [28], Flask 2.0, and Numpy 1.22 libraries. PacVIEW was built implementing NGL viewer [41,42,43], a web-based molecular graphics for large complexes, supported by the most widely used browsers. The website is written in HTML 5 and PHP 7.4. For all the PacDOCK tools, there is a limit of 50 MB on input file dimensions.

## Data Availability

PacDOCK is available at https://pegasus.lbic.unibo.it/pacdock. The input files used for the comparison with similar web servers are freely available at https://site.unibo.it/bioinorgchem/en/downloads.

## Supplementary Materials

The following supporting information can be downloaded at https://www.mdpi.com/article/10.3390/molecules27206884/s1. Section S1: Additional materials and methods; Section S2: Usage example: 5CGC; Section S3: Usage example: 1DNE; Section S4: Usage example: 6DDF; Section S5: Comparison with similar web servers.
