# Supplementary material for: PacDOCK: A Web Server for Positional Distance-Based and Interaction-Based Analysis of Docking Results
Source: Molecules. 2022 Oct 14;27(20):6884. doi: 10.3390/molecules27206884 (PMC9610523; doi:10.3390/molecules27206884)
Supplement: Supplementary file 1 [file molecules-27-06884-s001.zip › molecules-1908741-supplementary.pdf]

# PacDOCK: a web server for the comparison and the clustering of molecular docking results

Jacopo Carbone[1,2,†], Alessia Ghidini[1,2,†], Antonio Romano[3], Luca Gentilucci[1,*] and Francesco Musiani[2,*]

## SUPPLEMENTARY DATA

**S1 ADDITIONAL MATERIALS AND METHODS**

**S2 USAGE EXAMPLE: 5CGC**

**S3 USAGE EXAMPLE: 1DNE**

**S4 USAGE EXAMPLE: 6DDF**

**S5 COMPARISON WITH SIMILAR WEB SERVERS**

## S1 ADDITIONAL MATERIALS AND METHODS

All the re-dockings were computed with OpenEye programs: MakeReceptor, OMEGA, and HYBRID [1].

The receptor files were downloaded from RCSB PDB [2] and set up using MakeReceptor. The docking box enclosing the active site was determined by creating an initial box around the bound ligand and then extending each side of the box by 5 Å around the ligand.

The ligand of the structures 5CGC (51D) and 1DNE (netropsin) were downloaded in SDF format from RSCB PDB, while the ligand of the structure 6DDF (DAMGO) and the other ligands used for comparison with other web servers were extracted from the complex structure using MakeReceptor.

For 51D, netropsin, and DAMGO, a file containing 10000 ligand conformers was generated using OMEGA with the mode “pose”. The minimum Root Mean Square Cartesian distance below which two conformers are duplicates (RMS) was set to 0.1 Å. For the ligand used for comparison with other web servers, the same software was used to generate 1000 conformers with an RMS of 0.5 Å.

The three docking calculations in the case of 51D, netropsin, and DAMGO were run using HYBRID, generating 10000 docked poses for each ligand with a docking resolution set to “High” (translational step-size of 1.0 Å and rotational step-size of 1.0 Å). For the other ligands, 100 poses per ligand were generated and the resolution was set to “Standard” (translational step-size of 1.0 Å and rotational step-size of 1.5 Å).

Regarding the clustering cut-off selection in the following examples, the commonly accepted cut-off of 2.0 Å may be useful in the case of flexible ligands. On the other hand, in the case of more rigid ligands the use of such a cut-off can produce only one cluster. Therefore, it may be useful to choose a smaller cut-off (1.0 or 0.5 Å in our examples) to better distinguish between the various poses.

## S2 USAGE EXAMPLE: 5CGC

ProRMSD performs the RMSD calculation after matching the atoms of the ligand in the experimental pose and the re-docked pose on the basis of the atom type, the atoms to which they are bound, and the bond order.

Table S1. Atom indexes for the experimental and re-docked poses of the ligand.

| Experimental pose | | | | | Re-docked pose | | | | |
|---|---|---|---|---|---|---|---|---|---|
| Atom index | | Coordinates (x, y, z) | | | Atom index | | Coordinates (x, y, z) | | |
| 1 | Cl | -24.1790 | 20.8910 | 44.1790 | 1 | C | -21.0621 | 18.4565 | 47.2079 |
| 2 | F | -25.0390 | 18.2510 | 43.3230 | 2 | C | -25.9419 | 13.3724 | 39.3465 |
| 3 | C | -24.0300 | 18.1900 | 44.2130 | 3 | C | -22.4757 | 17.1703 | 45.6821 |
| 4 | C | -23.5200 | 19.3770 | 44.7100 | 4 | C | -22.6665 | 19.5780 | 45.7416 |
| 5 | C | -22.4900 | 19.3650 | 45.6240 | 5 | C | -24.3337 | 15.7216 | 42.8659 |
| 6 | C | -21.9580 | 18.1510 | 46.0550 | 6 | C | -25.7355 | 14.7591 | 39.2197 |
| 7 | C | -22.4660 | 16.9530 | 45.5590 | 7 | C | -25.5596 | 13.0846 | 40.6340 |
| 8 | C | -23.5090 | 16.9640 | 44.6400 | 8 | C | -24.1563 | 13.6085 | 44.5132 |
| 9 | C | -24.0150 | 15.6600 | 44.1290 | 9 | C | -22.0765 | 18.4012 | 46.2028 |
| 10 | C | -24.4050 | 15.5000 | 42.7970 | 10 | C | -23.4648 | 17.1162 | 44.7000 |
| 11 | C | -24.8580 | 14.2550 | 42.3780 | 11 | C | -24.0548 | 18.2930 | 44.2389 |
| 12 | N | -24.8980 | 13.2540 | 43.2510 | 12 | C | -23.6557 | 19.5239 | 44.7597 |
| 13 | C | -24.5130 | 13.4360 | 44.4990 | 13 | C | -23.8754 | 15.8368 | 44.1625 |
| 14 | N | -24.0830 | 14.6030 | 44.9380 | 14 | C | -24.6948 | 14.4496 | 42.4712 |
| 15 | N | -25.2490 | 14.0660 | 41.0520 | 15 | N | -20.2383 | 18.5014 | 48.0241 |
| 16 | C | -25.5550 | 12.8780 | 40.4720 | 16 | N | -25.2592 | 15.3139 | 40.3375 |
| 17 | C | -25.8690 | 13.1270 | 39.1830 | 17 | N | -23.7733 | 14.7974 | 45.0166 |
| 18 | C | -25.7500 | 14.5050 | 38.9610 | 18 | N | -24.6185 | 13.3662 | 43.2717 |
| 19 | N | -25.3770 | 15.0660 | 40.0800 | 19 | N | -25.1633 | 14.2704 | 41.1789 |
| 20 | C | -20.8890 | 18.1419 | 47.0030 | 20 | F | -25.0062 | 18.2416 | 43.2930 |
| 21 | N | -20.0410 | 18.1570 | 47.7560 | 21 | Cl | -24.3787 | 20.9806 | 44.1940 |

Table S2. Atom matching between ligand atoms in the experimental pose and the re-docked pose.

| Experimental pose | | | | | Re-docked pose | | | | |
|---|---|---|---|---|---|---|---|---|---|
| Atom index | | Coordinates (x, y, z) | | | Atom index | | Coordinates (x, y, z) | | |
| 1 | Cl | -24.1790 | 20.8910 | 44.1790 | 21 | Cl | -24.3787 | 20.9806 | 44.1940 |
| 2 | F | -25.0390 | 18.2510 | 43.3230 | 20 | F | -25.0062 | 18.2416 | 43.2930 |
| 3 | C | -24.0300 | 18.1900 | 44.2130 | 11 | C | -24.0548 | 18.2930 | 44.2389 |
| 4 | C | -23.5200 | 19.3770 | 44.7100 | 12 | C | -23.6557 | 19.5239 | 44.7597 |
| 5 | C | -22.4900 | 19.3650 | 45.6240 | 4 | C | -22.6665 | 19.5780 | 45.7416 |
| 6 | C | -21.9580 | 18.1510 | 46.0550 | 9 | C | -22.0765 | 18.4012 | 46.2028 |
| 7 | C | -22.4660 | 16.9530 | 45.5590 | 3 | C | -22.4757 | 17.1703 | 45.6821 |
| 8 | C | -23.5090 | 16.9640 | 44.6400 | 10 | C | -23.4648 | 17.1162 | 44.7000 |
| 9 | C | -24.0150 | 15.6600 | 44.1290 | 13 | C | -23.8754 | 15.8368 | 44.1625 |
| 10 | C | -24.4050 | 15.5000 | 42.7970 | 5 | C | -24.3337 | 15.7216 | 42.8659 |
| 11 | C | -24.8580 | 14.2550 | 42.3780 | 14 | C | -24.6948 | 14.4496 | 42.4712 |
| 12 | N | -24.8980 | 13.2540 | 43.2510 | 18 | N | -24.6185 | 13.3662 | 43.2717 |
| 13 | C | -24.5130 | 13.4360 | 44.4990 | 8 | C | -24.1563 | 13.6085 | 44.5132 |
| 14 | N | -24.0830 | 14.6030 | 44.9380 | 17 | N | -23.7733 | 14.7974 | 45.0166 |
| 15 | N | -25.2490 | 14.0660 | 41.0520 | 19 | N | -25.1633 | 14.2704 | 41.1789 |
| 16 | C | -25.5550 | 12.8780 | 40.4720 | 7 | C | -25.5596 | 13.0846 | 40.6340 |
| 17 | C | -25.8690 | 13.1270 | 39.1830 | 2 | C | -25.9419 | 13.3724 | 39.3465 |
| 18 | C | -25.7500 | 14.5050 | 38.9610 | 6 | C | -25.7355 | 14.7591 | 39.2197 |
| 19 | N | -25.3770 | 15.0660 | 40.0800 | 16 | N | -25.2592 | 15.3139 | 40.3375 |
| 20 | C | -20.8890 | 18.1419 | 47.0030 | 1 | C | -21.0621 | 18.4565 | 47.2079 |
| 21 | N | -20.0410 | 18.1570 | 47.7560 | 15 | N | -20.2383 | 18.5014 | 48.0241 |

Table S3. RMSD values for the re-docked poses of the ligand with respect to the experimental pose calculated by using ProRMSD and two similar web servers: DockRMSD and LigRMSD [3, 4].

| | **ProRMSD** | **DockRMSD** | **LigRMSD** |
|---|---|---|---|
| **RMSD** | 0.297 | 0.297 | 0.30 |

Note: RMSD value for the web server LigRMSD was reported with two decimal digits, as this is how the value is reported by the web server.

ClusDOCK performs the cluster analysis with three different algorithms. In this example, the gromos algorithm was employed, with a cut-off of 0.5 Å and a minimum cluster size of 1 structure. The steps that lead to cluster formations are illustrated below, in Table S4-S5.

Table S4. Pairwise RMSD matrix (10×10) in which each element contains the RMSD of the $i^{th}$ binding pose calculated with respect to the $j^{th}$ binding pose of the re-docking. Considering a cut-off of 0.5 Å, the number of neighbours is calculated (RMSD values below the cut-off are highlighted in gold). Structures 1 and 3 have the highest number of neighbours, i.e., six. Among these, the first pose (highlighted in green) has the best scoring function, thus it represents the centre of the most populated cluster, and forms together with all its neighbours (poses no. 2, 3, 4, 6, 8, and 9; highlighted in yellow) a cluster. Therefore, the first cluster is composed of structures 1, 2, 3, 4, 6, 8, and 9, and presents the scoring function of structure 1, the centroid of the cluster. These structures are thereafter eliminated from the pool of structures.

| Pose no. | 1 | 2 | 3 | 4 | 5 | 6 | 7 | 8 | 9 | 10 |
|---|---|---|---|---|---|---|---|---|---|---|
| 1 | 0 | 0.478 | 0.393 | 0.350 | 0.750 | 0.485 | 2.682 | 0.361 | 0.403 | 1.048 |
| 2 | 0.478 | 0 | 0.290 | 0.472 | 0.665 | 0.302 | 2.662 | 0.531 | 0.606 | 0.907 |
| 3 | 0.393 | 0.290 | 0 | 0.286 | 0.614 | 0.427 | 2.650 | 0.362 | 0.414 | 0.859 |
| 4 | 0.350 | 0.472 | 0.286 | 0 | 0.566 | 0.505 | 2.669 | 0.352 | 0.307 | 0.835 |
| 5 | 0.750 | 0.665 | 0.614 | 0.566 | 0 | 0.734 | 2.711 | 0.689 | 0.664 | 0.512 |
| 6 | 0.485 | 0.302 | 0.427 | 0.505 | 0.734 | 0 | 2.640 | 0.523 | 0.581 | 0.893 |
| 7 | 2.682 | 2.662 | 2.650 | 2.669 | 2.711 | 2.640 | 0 | 2.653 | 2.661 | 2.743 |
| 8 | 0.361 | 0.531 | 0.362 | 0.352 | 0.689 | 0.523 | 2.653 | 0 | 0.143 | 0.869 |
| 9 | 0.403 | 0.606 | 0.414 | 0.307 | 0.664 | 0.581 | 2.661 | 0.143 | 0 | 0.844 |
| 10 | 1.048 | 0.907 | 0.859 | 0.835 | 0.512 | 0.893 | 2.743 | 0.869 | 0.844 | 0 |
| No. of neighbours | 6 | 4 | 6 | 5 | 0 | 3 | 0 | 4 | 4 | 0 |

Table S5. Pairwise RMSD matrix (3×3) recalculated after the elimination of the first cluster structures. The values of RMSD calculated between the three remaining structures are higher than the cut-off, thus they remain as singleton clusters, each one with its relative scoring function.

| Pose no. | 5 | 7 | 10 |
|---|---|---|---|
| 5 | 0 | 2.711 | 0.512 |
| 7 | 2.711 | 0 | 2.743 |
| 10 | 0.512 | 2.743 | 0 |
| No. of neighbours | 0 | 0 | 0 |

Figure S1. Result of cluster analysis with the gromos algorithm on 51D re-docking results. (A) For each cluster is reported the cluster population, the structure indexes, and the scoring function of the centroid of the cluster that was selected as the most representative structure. The order of the clusters depends on their scoring function (the first one has the lowest scoring function). (B) Bar plot that shows on the y-axis the population for each cluster and on the x-axis the scoring function of the centroid of each cluster. The representative structure of each cluster is also reported.

| CLUSTER | #CONFORMATIONS | STRUCTURES | SCORING FUNCTION |
|---|---|---|---|
| 1 | 7 | 1, 2, 3, 4, 6, 8, 9 | -17.532913 |
| 2 | 1 | 5 | -16.862759 |
| 3 | 1 | 7 | -16.757294 |
| 4 | 1 | 10 | -16.584923 |

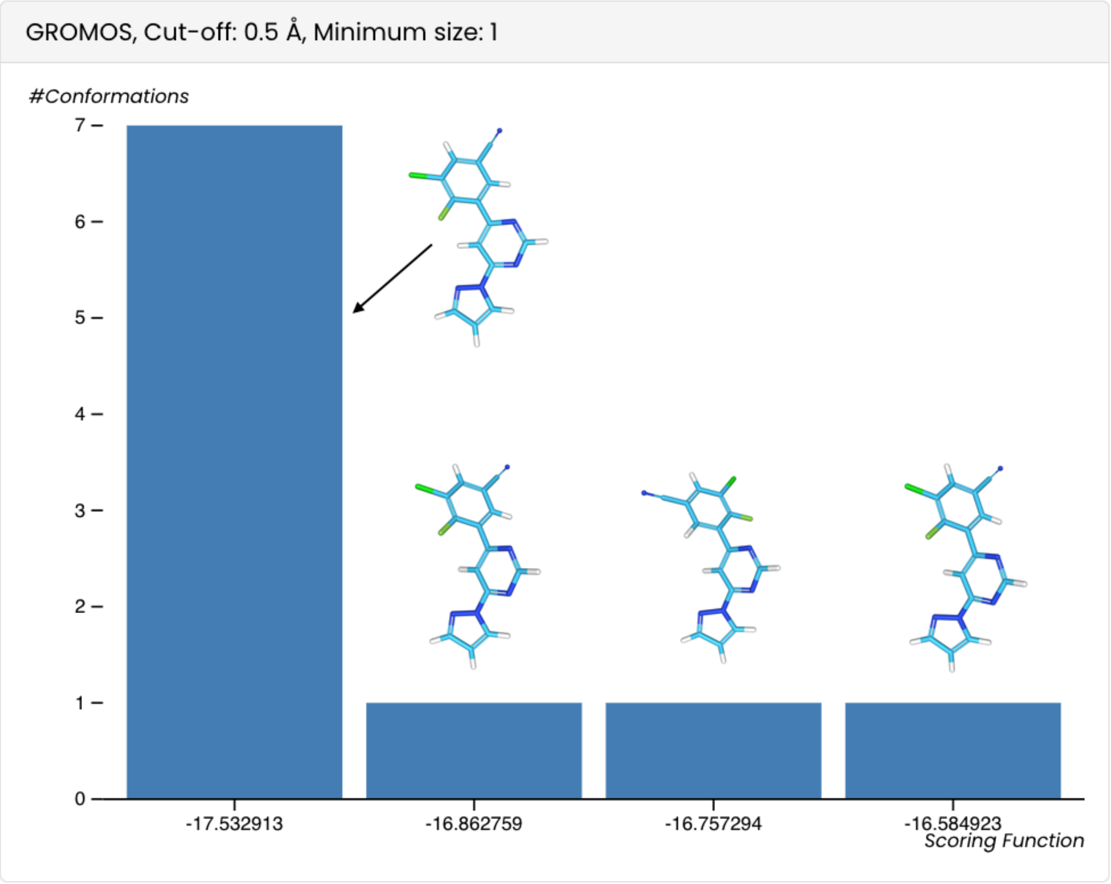

Figure S2. Result of cluster analysis with the single-linkage algorithm on 51D re-docking results. (A) For each cluster is reported the cluster population, the structure indexes, and the scoring function of the centroid of the cluster that was selected as the most representative structure. The order of the clusters depends on their scoring function (the first one has the lowest scoring function). (B) Bar plot that shows on the y-axis the population for each cluster and on the x-axis the scoring function of the centroid of each cluster. The representative structure of each cluster is also reported.

| CLUSTER | #CONFORMATIONS | STRUCTURES | SCORING FUNCTION |
|---|---|---|---|
| 1 | 7 | 1, 2, 3, 4, 6, 8, 9 | -17.532913 |
| 2 | 1 | 5 | -16.862759 |
| 3 | 1 | 7 | -16.757294 |
| 4 | 1 | 10 | -16.584923 |

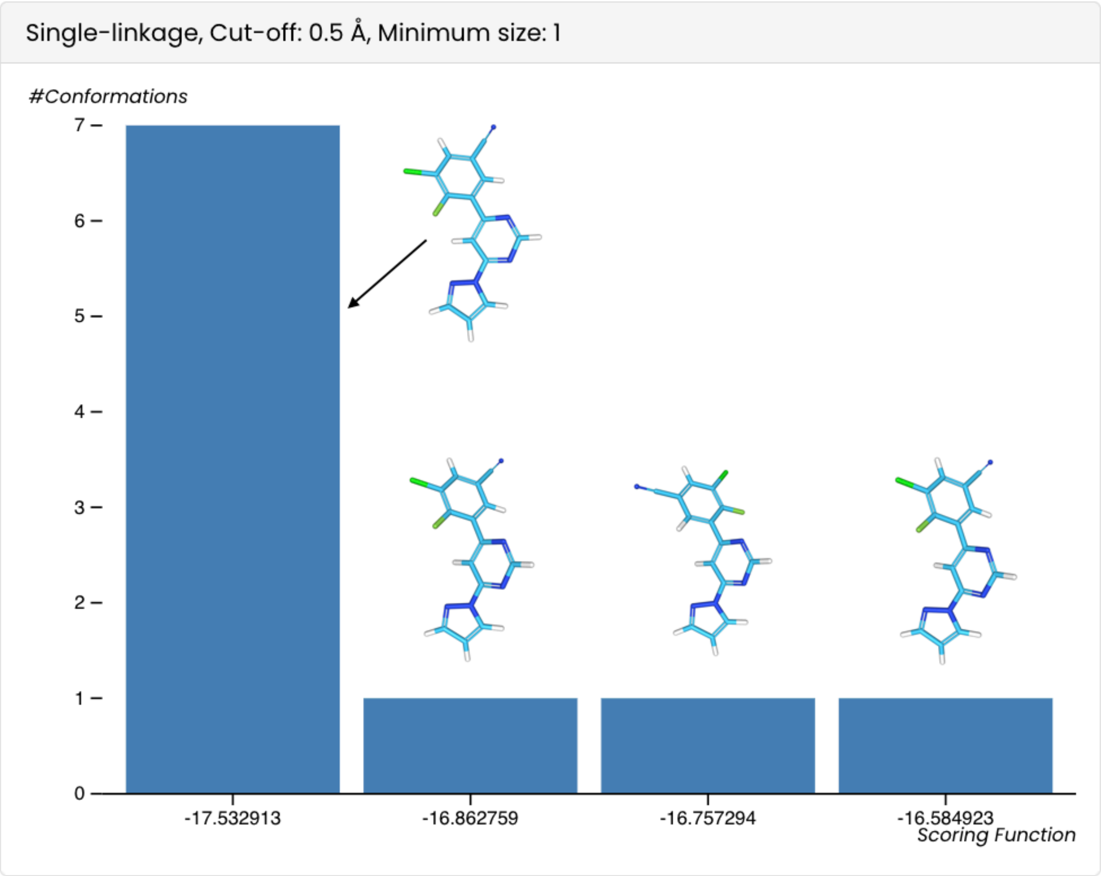

Figure S3. Result of cluster analysis with the complete-linkage algorithm on 51D re-docking results. (A) For each cluster is reported the cluster population, the structure indexes, and the scoring function of the centroid of the cluster that was selected as the most representative structure. The order of the clusters depends on their scoring function (the first one has the lowest scoring function). (B) Bar plot that shows on the y-axis the population for each cluster and on the x-axis the scoring function of the centroid of each cluster. The representative structure of each cluster is also reported.

| CLUSTER | #CONFORMATIONS | STRUCTURES | SCORING FUNCTION |
|---|---|---|---|
| 1 | 5 | 1, 3, 4, 8, 9 | -17.532913 |
| 2 | 2 | 2, 6 | -17.053669 |
| 3 | 1 | 5 | -16.862759 |
| 4 | 1 | 7 | -16.757294 |
| 5 | 1 | 10 | -16.584923 |

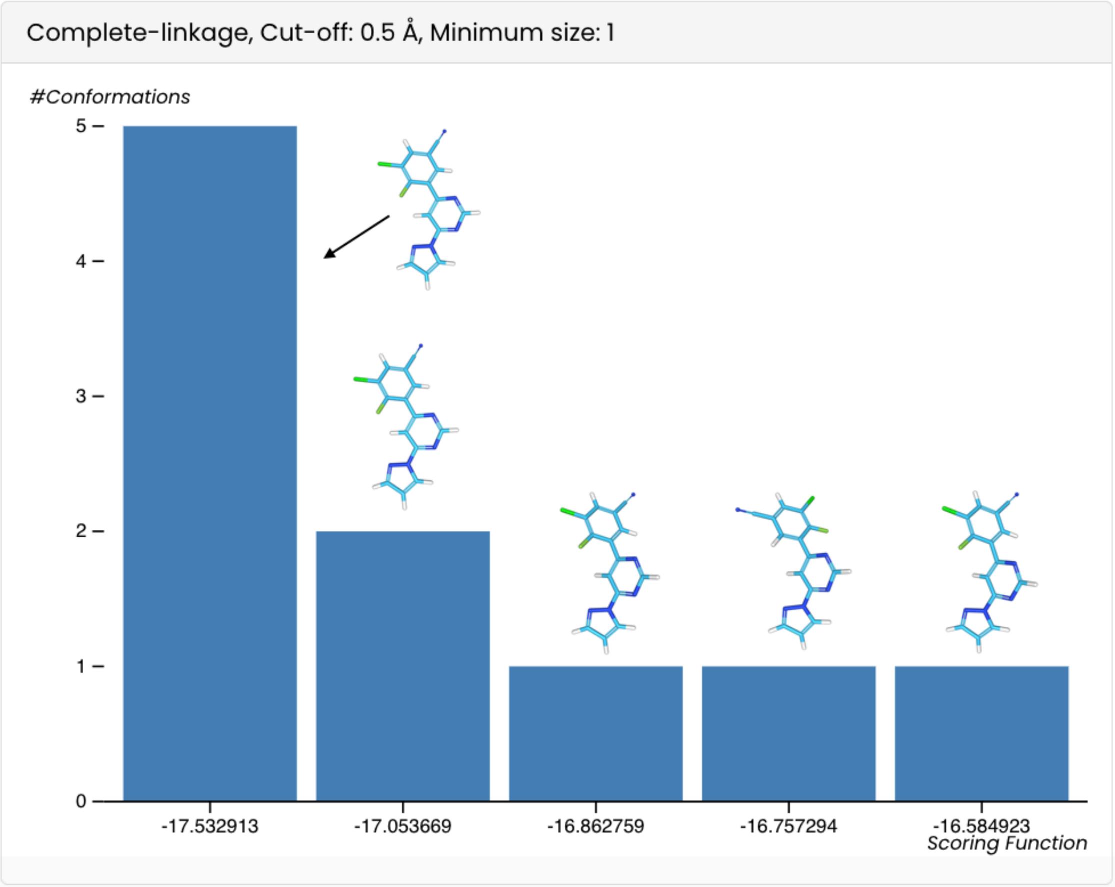


In this example, the use of the gromos and single-linkage algorithms returns the same clusters, while the complete-linkage algorithm allows one more cluster to be identified by splitting the most populated cluster obtained in the other cases into two smaller assemblies.

## S3 USAGE EXAMPLE: 1DNE

ProRMSD performs the RMSD calculation after matching the atoms of the ligand in the experimental pose and the re-docked pose on the basis of the atom type, the atoms to which they are bound, and the bond order.

Table S6. Atom indexes for the experimental and re-docked poses of netropsin.

| Experimental pose | | | | | Re-docked pose | | | | |
|---|---|---|---|---|---|---|---|---|---|
| Atom index | | Coordinates (x, y, z) | | | Atom index | | Coordinates (x, y, z) | | |
| 1 | C | 12.778 | 25.090 | 69.997 | 1 | C | 9.900 | 21.094 | 78.437 |
| 2 | N | 12.564 | 23.865 | 69.582 | 2 | C | 10.534 | 23.415 | 73.774 |
| 3 | N | 13.724 | 25.870 | 69.495 | 3 | C | 7.776 | 21.699 | 77.997 |
| 4 | N | 11.932 | 25.495 | 70.926 | 4 | C | 9.901 | 25.319 | 72.753 |
| 5 | C | 11.942 | 26.559 | 71.802 | 5 | C | 9.072 | 21.819 | 77.545 |
| 6 | C | 10.694 | 26.076 | 72.652 | 6 | C | 10.859 | 24.329 | 72.741 |
| 7 | O | 9.764 | 26.699 | 72.217 | 7 | C | 9.086 | 20.552 | 79.406 |
| 8 | N | 10.763 | 24.979 | 73.300 | 8 | C | 9.388 | 23.873 | 74.384 |
| 9 | C | 9.797 | 24.401 | 74.177 | 9 | C | 8.664 | 23.261 | 75.511 |
| 10 | C | 10.169 | 23.638 | 75.260 | 10 | C | 9.489 | 19.709 | 80.561 |
| 11 | C | 9.058 | 23.320 | 75.956 | 11 | C | 12.597 | 17.172 | 82.844 |
| 12 | N | 7.991 | 23.902 | 75.327 | 12 | C | 12.279 | 25.161 | 70.834 |
| 13 | C | 6.546 | 23.869 | 75.661 | 13 | C | 14.986 | 25.514 | 68.234 |
| 14 | C | 8.464 | 24.620 | 74.250 | 14 | C | 6.627 | 20.569 | 79.901 |
| 15 | C | 8.757 | 22.437 | 77.093 | 15 | C | 7.857 | 25.838 | 74.085 |
| 16 | O | 7.555 | 22.157 | 77.313 | 16 | C | 12.054 | 17.946 | 81.702 |
| 17 | N | 9.698 | 21.901 | 77.868 | 17 | C | 13.538 | 24.717 | 70.115 |
| 18 | C | 9.308 | 20.948 | 78.918 | 18 | C | 11.304 | 19.187 | 82.170 |
| 19 | C | 10.189 | 20.461 | 79.908 | 19 | N | 7.797 | 20.926 | 79.129 |
| 20 | C | 9.491 | 19.561 | 80.697 | 20 | N | 9.012 | 25.031 | 73.755 |
| 21 | N | 8.199 | 19.491 | 80.122 | 21 | N | 13.837 | 17.431 | 83.233 |
| 22 | C | 7.071 | 18.674 | 80.537 | 22 | N | 15.260 | 26.451 | 67.244 |
| 23 | C | 8.118 | 20.316 | 79.072 | 23 | N | 9.461 | 22.523 | 76.429 |
| 24 | C | 9.843 | 18.641 | 81.727 | 24 | N | 11.925 | 24.266 | 71.875 |
| 25 | O | 9.053 | 17.837 | 82.249 | 25 | N | 10.761 | 19.938 | 81.071 |
| 26 | N | 11.191 | 18.352 | 81.915 | 26 | N | 13.867 | 25.658 | 69.075 |
| 27 | C | 11.390 | 17.251 | 82.965 | 27 | N | 11.808 | 16.267 | 83.405 |
| 28 | C | 12.707 | 17.156 | 83.587 | 28 | N | 15.833 | 24.422 | 68.389 |
| 29 | C | 12.822 | 16.987 | 85.085 | 29 | O | 7.444 | 23.368 | 75.612 |
| 30 | N | 11.790 | 17.016 | 85.907 | 30 | O | 8.710 | 18.859 | 80.984 |
| 31 | N | 14.011 | 16.822 | 85.653 | 31 | O | 11.676 | 26.184 | 70.527 |

Table S7. Atom matching between netropsin atoms in the experimental pose and the re-docked pose.

| Experimental pose | | | | | Re-docked pose | | | | |
|---|---|---|---|---|---|---|---|---|---|
| Atom index | | Coordinates (x, y, z) | | | Atom index | | Coordinates (x, y, z) | | |
| 1 | C | 12.778 | 25.090 | 69.997 | 13 | C | 14.986 | 25.514 | 68.234 |
| 2 | N | 12.564 | 23.865 | 69.582 | 28 | N | 15.833 | 24.422 | 68.389 |
| 3 | N | 13.724 | 25.870 | 69.495 | 22 | N | 15.260 | 26.451 | 67.244 |
| 4 | N | 11.932 | 25.495 | 70.926 | 26 | N | 13.867 | 25.658 | 69.075 |
| 5 | C | 11.942 | 26.559 | 71.802 | 17 | C | 13.538 | 24.717 | 70.115 |
| 6 | C | 10.694 | 26.076 | 72.652 | 12 | C | 12.279 | 25.161 | 70.834 |
| 7 | O | 9.764 | 26.699 | 72.217 | 31 | O | 11.676 | 26.184 | 70.527 |
| 8 | N | 10.763 | 24.979 | 73.300 | 24 | N | 11.925 | 24.266 | 71.875 |
| 9 | C | 9.797 | 24.401 | 74.177 | 6 | C | 10.859 | 24.329 | 72.741 |
| 10 | C | 10.169 | 23.638 | 75.260 | 2 | C | 10.534 | 23.415 | 73.774 |
| 11 | C | 9.058 | 23.320 | 75.956 | 8 | C | 9.388 | 23.873 | 74.384 |
| 12 | N | 7.991 | 23.902 | 75.327 | 20 | N | 9.012 | 25.031 | 73.755 |
| 13 | C | 6.546 | 23.869 | 75.661 | 15 | C | 7.857 | 25.838 | 74.085 |
| 14 | C | 8.464 | 24.620 | 74.250 | 4 | C | 9.901 | 25.319 | 72.753 |
| 15 | C | 8.757 | 22.437 | 77.093 | 9 | C | 8.664 | 23.261 | 75.511 |
| 16 | O | 7.555 | 22.157 | 77.313 | 29 | O | 7.444 | 23.368 | 75.612 |
| 17 | N | 9.698 | 21.901 | 77.868 | 23 | N | 9.461 | 22.523 | 76.429 |
| 18 | C | 9.308 | 20.948 | 78.918 | 5 | C | 9.072 | 21.819 | 77.545 |
| 19 | C | 10.189 | 20.461 | 79.908 | 1 | C | 9.900 | 21.094 | 78.437 |
| 20 | C | 9.491 | 19.561 | 80.697 | 7 | C | 9.086 | 20.552 | 79.406 |
| 21 | N | 8.199 | 19.491 | 80.122 | 19 | N | 7.797 | 20.926 | 79.129 |
| 22 | C | 7.071 | 18.674 | 80.537 | 14 | C | 6.627 | 20.569 | 79.901 |
| 23 | C | 8.118 | 20.316 | 79.072 | 3 | C | 7.776 | 21.699 | 77.997 |
| 24 | C | 9.843 | 18.641 | 81.727 | 10 | C | 9.489 | 19.709 | 80.561 |
| 25 | O | 9.053 | 17.837 | 82.249 | 30 | O | 8.710 | 18.859 | 80.984 |
| 26 | N | 11.191 | 18.352 | 81.915 | 25 | N | 10.761 | 19.938 | 81.071 |
| 27 | C | 11.390 | 17.251 | 82.965 | 18 | C | 11.304 | 19.187 | 82.170 |
| 28 | C | 12.707 | 17.156 | 83.587 | 16 | C | 12.054 | 17.946 | 81.702 |
| 29 | C | 12.822 | 16.987 | 85.085 | 11 | C | 12.597 | 17.172 | 82.844 |
| 30 | N | 11.790 | 17.016 | 85.907 | 27 | N | 11.808 | 16.267 | 83.405 |
| 31 | N | 14.011 | 16.822 | 85.653 | 21 | N | 13.837 | 17.431 | 83.233 |

Table S8. RMSD values for the re-docked poses of netropsin with respect to the experimental pose calculated by using ProRMSD, DockRMSD, and LigRMSD.

| | ProRMSD | DockRMSD | LigRMSD |
|---|---|---|---|
| **RMSD** | 2.219 | 2.219 | 2.22 |

Note: RMSD value for the web server LigRMSD was reported with two decimal digits, as this is how the value is reported by the web server.

ClusDOCK performs the cluster analysis with three different algorithms. In this example, the gromos algorithm was employed, with a cut-off of 1.0 Å and a minimum cluster size of 1 structure. The steps that lead to cluster formations are illustrated below, in Table S9-S14.

Table S9. Pairwise RMSD matrix (20×20) in which each element contains the RMSD of the $i^{th}$ binding pose calculated with respect to the $j^{th}$ binding pose of netropsin re-docking. Considering a cut-off of 1.0 Å, the number of neighbours is calculated (RMSD values below the cut-off are highlighted in gold). Structures 3, 6, and 14 have the highest number of neighbours, i.e., five. Among these, the third pose (highlighted in green) has the best scoring function, thus it represents the centre of the most populated cluster, and forms together with all its neighbours (poses no. 5, 6, 8, 16, and 17; highlighted in yellow) a cluster. Therefore, the first cluster is composed of structures 3, 5, 6, 8, 16, and 17, and presents the scoring function of structure 3, the centroid of the cluster. These structures are thereafter eliminated from the pool of structures.

| Pose no. | 1 | 2 | 3 | 4 | 5 | 6 | 7 | 8 | 9 | 10 | 11 | 12 | 13 | 14 | 15 | 16 | 17 | 18 | 19 | 20 |
|---|---|---|---|---|---|---|---|---|---|---|---|---|---|---|---|---|---|---|---|---|
| 1 | 0 | 0.166 | 11.600 | 3.411 | 11.609 | 11.607 | 4.079 | 11.602 | 3.623 | 0.661 | 1.147 | 4.136 | 1.022 | 0.933 | 3.724 | 11.639 | 11.612 | 1.101 | 11.426 | 11.449 |
| 2 | 0.166 | 0 | 11.602 | 3.435 | 11.606 | 11.612 | 4.096 | 11.604 | 3.650 | 0.675 | 1.128 | 4.162 | 1.032 | 0.923 | 3.749 | 11.650 | 11.612 | 1.039 | 11.422 | 11.446 |
| 3 | 11.600 | 11.602 | 0 | 11.732 | 0.629 | 0.400 | 11.413 | 0.479 | 11.787 | 11.614 | 11.807 | 11.432 | 11.797 | 11.802 | 11.789 | 0.910 | 0.804 | 11.819 | 1.275 | 1.424 |
| 4 | 3.411 | 3.435 | 11.732 | 0 | 11.794 | 11.723 | 1.696 | 11.775 | 0.789 | 3.303 | 3.110 | 1.731 | 3.139 | 3.373 | 2.155 | 11.711 | 11.696 | 3.583 | 11.589 | 11.550 |
| 5 | 11.609 | 11.606 | 0.629 | 11.794 | 0 | 0.854 | 11.502 | 0.350 | 11.873 | 11.628 | 11.810 | 11.531 | 11.808 | 11.798 | 11.803 | 1.353 | 0.764 | 11.796 | 1.065 | 1.304 |
| 6 | 11.607 | 11.612 | 0.400 | 11.723 | 0.854 | 0 | 11.383 | 0.588 | 11.763 | 11.605 | 11.809 | 11.406 | 11.799 | 11.808 | 11.763 | 0.559 | 0.775 | 11.833 | 1.382 | 1.463 |
| 7 | 4.079 | 4.096 | 11.413 | 1.696 | 11.502 | 11.383 | 0 | 11.473 | 1.476 | 3.814 | 3.713 | 0.393 | 3.802 | 4.079 | 3.041 | 11.337 | 11.340 | 4.294 | 11.268 | 11.178 |
| 8 | 11.602 | 11.604 | 0.479 | 11.775 | 0.350 | 0.588 | 11.473 | 0 | 11.841 | 11.614 | 11.807 | 11.498 | 11.799 | 11.795 | 11.782 | 1.043 | 0.705 | 11.803 | 1.165 | 1.345 |
| 9 | 3.623 | 3.650 | 11.787 | 0.789 | 11.873 | 11.763 | 1.476 | 11.841 | 0 | 3.432 | 3.287 | 1.509 | 3.314 | 3.578 | 2.362 | 11.728 | 11.758 | 3.805 | 11.679 | 11.626 |
| 10 | 0.661 | 0.675 | 11.614 | 3.303 | 11.628 | 11.605 | 3.814 | 11.614 | 3.432 | 0 | 1.035 | 3.897 | 1.016 | 1.057 | 3.594 | 11.617 | 11.600 | 1.223 | 11.431 | 11.431 |
| 11 | 1.147 | 1.128 | 11.807 | 3.110 | 11.810 | 11.809 | 3.713 | 11.807 | 3.287 | 1.035 | 0 | 3.796 | 0.319 | 0.565 | 3.355 | 11.834 | 11.790 | 0.817 | 11.582 | 11.582 |
| 12 | 4.136 | 4.162 | 11.432 | 1.731 | 11.531 | 11.406 | 0.393 | 11.498 | 1.509 | 3.897 | 3.796 | 0 | 3.868 | 4.151 | 3.163 | 11.353 | 11.372 | 4.383 | 11.305 | 11.211 |
| 13 | 1.022 | 1.032 | 11.797 | 3.139 | 11.808 | 11.799 | 3.802 | 11.799 | 3.314 | 1.016 | 0.319 | 3.868 | 0 | 0.444 | 3.387 | 11.821 | 11.796 | 0.813 | 11.591 | 11.599 |
| 14 | 0.933 | 0.923 | 11.802 | 3.373 | 11.798 | 11.808 | 4.079 | 11.795 | 3.578 | 1.057 | 0.565 | 4.151 | 0.444 | 0 | 3.455 | 11.842 | 11.799 | 0.474 | 11.594 | 11.612 |
| 15 | 3.724 | 3.749 | 11.789 | 2.155 | 11.803 | 11.763 | 3.041 | 11.782 | 2.362 | 3.594 | 3.355 | 3.163 | 3.387 | 3.455 | 0 | 11.755 | 11.697 | 3.563 | 11.594 | 11.569 |
| 16 | 11.639 | 11.650 | 0.910 | 11.711 | 1.353 | 0.559 | 11.337 | 1.043 | 11.728 | 11.617 | 11.834 | 11.353 | 11.821 | 11.842 | 11.755 | 0 | 1.073 | 11.882 | 1.731 | 1.688 |
| 17 | 11.612 | 11.612 | 0.804 | 11.696 | 0.764 | 0.775 | 11.340 | 0.705 | 11.758 | 11.600 | 11.790 | 11.372 | 11.796 | 11.799 | 11.697 | 1.073 | 0 | 11.812 | 1.055 | 1.026 |
| 18 | 1.101 | 1.039 | 11.819 | 3.583 | 11.796 | 11.833 | 4.294 | 11.803 | 3.805 | 1.223 | 0.817 | 4.383 | 0.813 | 0.474 | 3.563 | 11.882 | 11.812 | 0 | 11.593 | 11.618 |
| 19 | 11.426 | 11.422 | 1.275 | 11.589 | 1.065 | 1.382 | 11.268 | 1.165 | 11.679 | 11.431 | 11.582 | 11.305 | 11.591 | 11.594 | 11.594 | 1.731 | 1.055 | 11.593 | 0 | 0.512 |
| 20 | 11.449 | 11.446 | 1.424 | 11.550 | 1.304 | 1.463 | 11.178 | 1.345 | 11.626 | 11.431 | 11.582 | 11.211 | 11.599 | 11.612 | 11.569 | 1.688 | 1.026 | 11.618 | 0.512 | 0 |
| No. of neighbours | 3 | 3 | 5 | 1 | 4 | 5 | 1 | 4 | 1 | 2 | 3 | 1 | 3 | 5 | 0 | 2 | 4 | 3 | 1 | 1 |

Table S10. Pairwise RMSD matrix (14×14) recalculated after the elimination of the first cluster structures. The structure 14 has the highest number of neighbours, thus it represents the centre of the new cluster, which includes its neighbours (poses no. 1, 2, 11, 13, and 18), and presents the scoring function of structure 14. These structures are thereafter eliminated from the pool of structures.

| Pose no. | 1 | 2 | 4 | 7 | 9 | 10 | 11 | 12 | 13 | 14 | 15 | 18 | 19 | 20 |
|---|---|---|---|---|---|---|---|---|---|---|---|---|---|---|
| 1 | 0 | 0.166 | 3.411 | 4.079 | 3.623 | 0.661 | 1.147 | 4.136 | 1.022 | 0.933 | 3.724 | 1.101 | 11.426 | 11.449 |
| 2 | 0.166 | 0 | 3.435 | 4.096 | 3.650 | 0.675 | 1.128 | 4.162 | 1.032 | 0.923 | 3.749 | 1.039 | 11.422 | 11.446 |
| 4 | 3.411 | 3.435 | 0 | 1.696 | 0.789 | 3.303 | 3.110 | 1.731 | 3.139 | 3.373 | 2.155 | 3.583 | 11.589 | 11.550 |
| 7 | 4.079 | 4.096 | 1.696 | 0 | 1.476 | 3.814 | 3.713 | 0.393 | 3.802 | 4.079 | 3.041 | 4.294 | 11.268 | 11.178 |
| 9 | 3.623 | 3.650 | 0.789 | 1.476 | 0 | 3.432 | 3.287 | 1.509 | 3.314 | 3.578 | 2.362 | 3.805 | 11.679 | 11.626 |
| 10 | 0.661 | 0.675 | 3.303 | 3.814 | 3.432 | 0 | 1.035 | 3.897 | 1.016 | 1.057 | 3.594 | 1.223 | 11.431 | 11.431 |
| 11 | 1.147 | 1.128 | 3.110 | 3.713 | 3.287 | 1.035 | 0 | 3.796 | 0.319 | 0.565 | 3.355 | 0.817 | 11.582 | 11.582 |
| 12 | 4.136 | 4.162 | 1.731 | 0.393 | 1.509 | 3.897 | 3.796 | 0 | 3.868 | 4.151 | 3.163 | 4.383 | 11.305 | 11.211 |
| 13 | 1.022 | 1.032 | 3.139 | 3.802 | 3.314 | 1.016 | 0.319 | 3.868 | 0 | 0.444 | 3.387 | 0.813 | 11.591 | 11.599 |
| 14 | 0.933 | 0.923 | 3.373 | 4.079 | 3.578 | 1.057 | 0.565 | 4.151 | 0.444 | 0 | 3.455 | 0.474 | 11.594 | 11.612 |
| 15 | 3.724 | 3.749 | 2.155 | 3.041 | 2.362 | 3.594 | 3.355 | 3.163 | 3.387 | 3.455 | 0 | 3.563 | 11.594 | 11.569 |
| 18 | 1.101 | 1.039 | 3.583 | 4.294 | 3.805 | 1.223 | 0.817 | 4.383 | 0.813 | 0.474 | 3.563 | 0 | 11.593 | 11.618 |
| 19 | 11.426 | 11.422 | 11.589 | 11.268 | 11.679 | 11.431 | 11.582 | 11.305 | 11.591 | 11.594 | 11.594 | 11.593 | 0 | 0.512 |
| 20 | 11.449 | 11.446 | 11.550 | 11.178 | 11.626 | 11.431 | 11.582 | 11.211 | 11.599 | 11.612 | 11.569 | 11.618 | 0.512 | 0 |
| No. of neighbours | 3 | 3 | 1 | 1 | 1 | 2 | 3 | 1 | 3 | 5 | 0 | 3 | 1 | 1 |

Table S11. Pairwise RMSD matrix (8×8) recalculated. Among the structures that have the highest number of neighbours, the number 4 has the best scoring function, thus it represents the centre of the new cluster, which includes its neighbour (pose no. 9), and presents the scoring function of structure 4. These structures are thereafter eliminated from the pool of structures.

| Pose no. | 4 | 7 | 9 | 10 | 12 | 15 | 19 | 20 |
|---|---|---|---|---|---|---|---|---|
| 4 | 0 | 1.696 | 0.789 | 3.303 | 1.731 | 2.155 | 11.589 | 11.550 |
| 7 | 1.696 | 0 | 1.476 | 3.814 | 0.393 | 3.041 | 11.268 | 11.178 |
| 9 | 0.789 | 1.476 | 0 | 3.432 | 1.509 | 2.362 | 11.679 | 11.626 |
| 10 | 3.303 | 3.814 | 3.432 | 0 | 3.897 | 3.594 | 11.431 | 11.431 |
| 12 | 1.731 | 0.393 | 1.509 | 3.897 | 0 | 3.163 | 11.305 | 11.211 |
| 15 | 2.155 | 3.041 | 2.362 | 3.594 | 3.163 | 0 | 11.594 | 11.569 |
| 19 | 11.589 | 11.268 | 11.679 | 11.431 | 11.305 | 11.594 | 0 | 0.512 |
| 20 | 11.550 | 11.178 | 11.626 | 11.431 | 11.211 | 11.569 | 0.512 | 0 |
| No. of neighbours | 1 | 1 | 1 | 0 | 1 | 0 | 1 | 1 |

Table S12. Pairwise RMSD matrix (6×6) recalculated. Among the structures that have the highest number of neighbours, the number 7 has the best scoring function, thus it represents the centre of the new cluster, which includes its neighbour (pose no. 12), and presents the scoring function of structure 7. These structures are thereafter eliminated from the pool of structures.

| Pose no. | 7 | 10 | 12 | 15 | 19 | 20 |
|---|---|---|---|---|---|---|
| 7 | 0 | 3.814 | 0.393 | 3.041 | 11.268 | 11.178 |
| 10 | 3.814 | 0 | 3.897 | 3.594 | 11.431 | 11.431 |
| 12 | 0.393 | 3.897 | 0 | 3.163 | 11.305 | 11.211 |
| 15 | 3.041 | 3.594 | 3.163 | 0 | 11.594 | 11.569 |
| 19 | 11.268 | 11.431 | 11.305 | 11.594 | 0 | 0.512 |
| 20 | 11.178 | 11.431 | 11.211 | 11.569 | 0.512 | 0 |
| No. of neighbours | 1 | 0 | 1 | 0 | 1 | 1 |

Table S13. Pairwise RMSD matrix (4×4) recalculated. Among the structures that have the highest number of neighbours, the number 19 has the best scoring function, thus it represents the centre of the new cluster, which includes its neighbour (pose no. 20), and presents the scoring function of structure 19. These structures are thereafter eliminated from the pool of structures.

| **Pose no.** | **10** | **15** | **19** | **20** |
|---|---|---|---|---|
| **10** | 0 | 3.594 | 11.431 | 11.431 |
| **15** | 3.594 | 0 | 11.594 | 11.569 |
| **19** | 11.431 | 11.594 | 0 | 0.512 |
| **20** | 11.431 | 11.569 | 0.512 | 0 |
| **No. of neighbours** | 0 | 0 | 1 | 1 |

Table S14. Pairwise RMSD matrix (2×2) recalculated. The value of RMSD calculated between the two remaining structures is higher than the cut-off, thus they remain as singleton clusters, each one with its relative scoring function.

| **Pose no.** | **10** | **15** |
|---|---|---|
| **10** | 0 | 3.594 |
| **15** | 3.594 | 0 |
| **No. of neighbours** | 0 | 0 |

Figure S4. Result of cluster analysis with the gromos algorithm on netropsin re-docking results. (A) For each cluster is reported the cluster population, the structure indexes, and the scoring function of the centroid of the cluster that was selected as the most representative structure. The order of the clusters depends on their scoring function (the first one has the lowest scoring function). (B) Bar plot that shows on the y-axis the population for each cluster and on the x-axis the scoring function of the centroid of each cluster. The representative structure of each cluster is also reported.

| CLUSTER | #CONFORMATIONS | STRUCTURES | SCORING FUNCTION |
|---|---|---|---|
| 1 | 6 | 3, 5, 6, 8, 16, 17 | -12.895995 |
| 2 | 2 | 4, 9 | -12.810198 |
| 3 | 2 | 7, 12 | -12.414389 |
| 4 | 1 | 10 | -12.01548 |
| 5 | 6 | 14, 1, 2, 11, 13, 18 | -11.732399 |
| 6 | 1 | 15 | -11.722769 |
| 7 | 2 | 19, 20 | -11.489742 |

GROMOS, Cut-off: 1 Å, Minimum size: 1

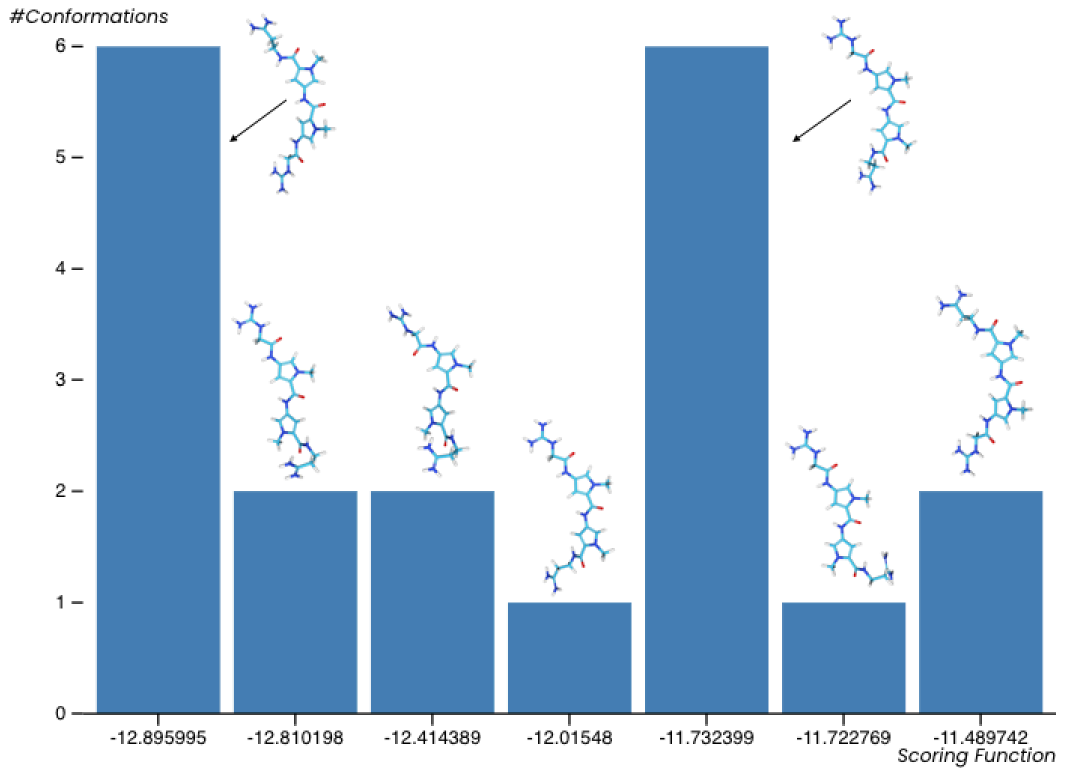

Figure S5. Result of cluster analysis with the single-linkage algorithm on netropsin re-docking results. (A) For each cluster is reported the cluster population, the structure indexes, and the scoring function of the centroid of the cluster that was selected as the most representative structure. The order of the clusters depends on their scoring function (the first one has the lowest scoring function). (B) Bar plot that shows on the y-axis the population for each cluster and on the x-axis the scoring function of the centroid of each cluster. The representative structure of each cluster is also reported.

| CLUSTER | #CONFORMATIONS | STRUCTURES | SCORING FUNCTION |
|---|---|---|---|
| 1 | 7 | 1, 2, 10, 11, 13, 14, 18 | -13.216396 |
| 2 | 6 | 3, 5, 6, 8, 16, 17 | -12.895995 |
| 3 | 2 | 4, 9 | -12.810198 |
| 4 | 2 | 7, 12 | -12.414389 |
| 5 | 1 | 15 | -11.722769 |
| 6 | 2 | 19, 20 | -11.489742 |

Single-linkage, Cut-off: 1 Å, Minimum size: 1

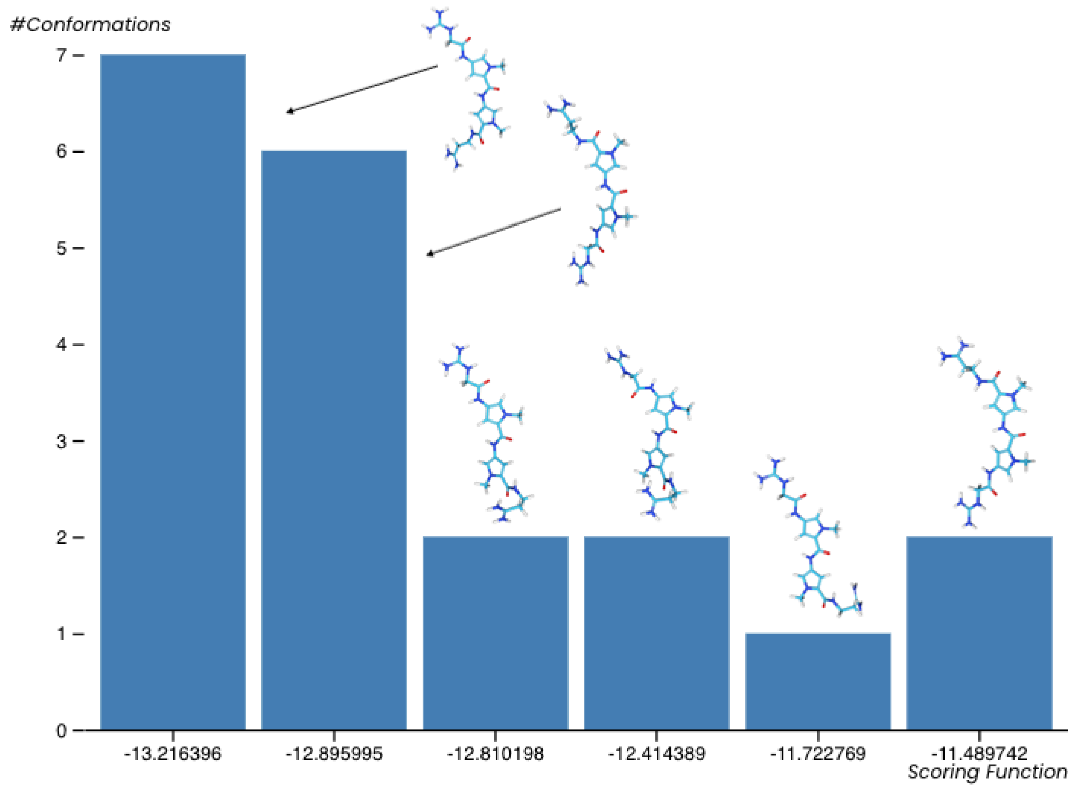

Figure S6. Result of cluster analysis with the complete-linkage algorithm on netropsin re-docking results. (A) For each cluster is reported the cluster population, the structure indexes, and the scoring function of the centroid of the cluster that was selected as the most representative structure. The order of the clusters depends on their scoring function (the first one has the lowest scoring function). (B) Bar plot that shows on the y-axis the population for each cluster and on the x-axis the scoring function of the centroid of each cluster. The representative structure of each cluster is also reported.

| CLUSTER | #CONFORMATIONS | STRUCTURES | SCORING FUNCTION |
|---|---|---|---|
| 1 | 3 | 1, 2, 10 | -13.216396 |
| 2 | 5 | 3, 5, 6, 8, 17 | -12.895995 |
| 3 | 2 | 4, 9 | -12.810198 |
| 4 | 2 | 7, 12 | -12.414389 |
| 5 | 4 | 11, 13, 14, 18 | -11.968822 |
| 6 | 1 | 15 | -11.722769 |
| 7 | 1 | 16 | -11.704092 |
| 8 | 2 | 19, 20 | -11.489742 |

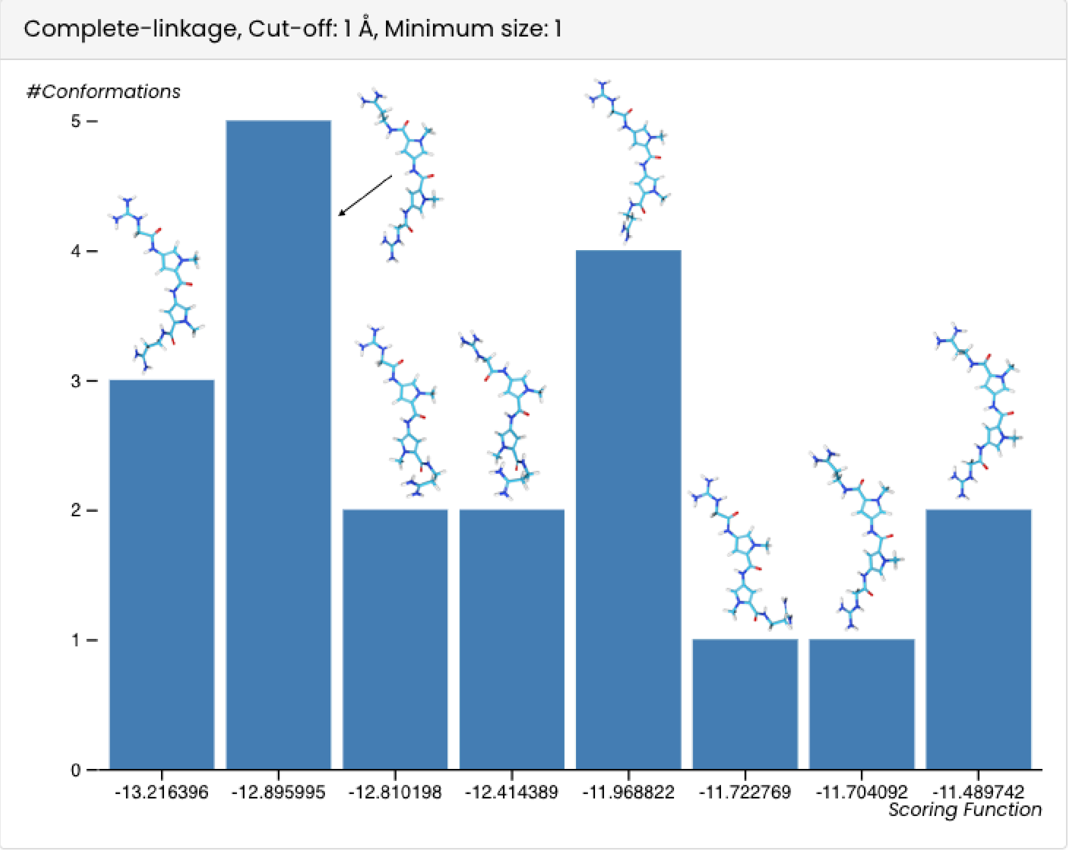

In this example, the three different algorithms produce a different number of clusters and a different population for each cluster. By using the gromos algorithm, the two largely populated clusters have the same number of binding poses, with the representative poses that are in one case very similar to the experimental one (pose #14, RMSD = 2.089 Å, slightly better than pose #1) and in the other case is completely upside-down with respect to the reference structure. The single-linkage and the complete-linkage algorithms appear to allow for a different distribution of the binding poses, with the single-linkage algorithm providing the most populated cluster (7 structures) able to include all the binding poses that are more similar to the experimental reference.

## S4 USAGE EXAMPLE: 6DDF

This section presents an example of PacDOCK usage for the complete analysis of docking results with RMSD calculation, visualisation of the docked pose with respect to the reference pose and receptor-ligand interactions, and clustering. This example regards the structure of the μ-opioid receptor (μOR)-$G_i$ protein complex bound to the agonist peptide DAMGO and nucleotide-free $G_i$ with a resolution of 3.5 Å (PDB ID: 6DDF). The μ-opioid receptor is a G-protein-coupled receptor (GPCR) and the target of most clinically and recreationally used opioids. The induced positive effects of analgesia and euphoria are mediated by μOR signalling through the adenylyl cyclase-inhibiting heterotrimeric G protein $G_i$ [5].

***ProRMSD***. Starting from the PDB file, DAMGO was separated from the receptor μOR, and then re-docked into the target using the docking program OpenEye HYBRID (*see Section S1)*. From the docking program output, the first pose of the ligand was used to create the input file to calculate the RMSD of this pose with respect to the experimental one by using ProRMSD with the default RMSD calculation mode. The atom matching procedure is required for the RMSD calculation since the atoms in the experimental and the docked pose are labelled differently (Figure S3). Through the use of ProRMSD, it is possible to calculate RMSD also in this case, where atoms are randomly labelled. The atomic order in the structure files before and after the matching is shown in Tables S15 and S16. The value of RMSD calculated between the reference and the docked pose is 1.973 Å. The RMSD calculation comparison with DockRMSD and LigRMSD is shown in Table S17.

***PacVIEW***. The same two structures used for the RMSD calculation with ProRMSD were visualised using PacVIEW in the Docking results mode. As shown in Figure S7, it is possible to visualise the spatial differences between the two poses of the ligand. Moreover, the ligand was displayed within the allosteric site of the receptor and the key interactions were investigated by using PacVIEW in the Receptor-Ligand interactions mode (Figure S3).

***ClusDOCK***. The first twenty poses from the re-docking were clustered with ClusDOCK by using the gromos algorithm, a cut-off of 1.0 Å, and a minimum cluster size of 1 structure (the pairwise RMSD matrix is reported in Table S18). The cluster analysis shows the grouping of the twenty poses in three different clusters. Interestingly, the first cluster, thus the most populated, is also the one with the most favourable scoring function. Additional information on the clustering can be found in Table S19 and the results are shown in Figures S8-S10.

ProRMSD performs the RMSD calculation after matching the atoms of the ligand in the experimental pose and the re-docked pose on the basis of the atom type, the atoms to which they are bound, and the bond order.

Table S15. Atom indexes for the experimental and re-docked poses of DAMGO.

| Experimental pose | | | | Re-docked pose | | | |
|---|---|---|---|---|---|---|---|
| Atom index | Coordinates (x, y, z) | | | Atom index | Coordinates (x, y, z) | | |
| 1 N | 119.032 | 149.353 | 141.168 | 1 C | 120.519 | 147.766 | 147.642 |
| 2 C | 119.627 | 150.658 | 140.911 | 2 C | 120.834 | 148.364 | 148.862 |
| 3 C | 118.634 | 151.725 | 141.330 | 3 C | 120.199 | 148.558 | 146.539 |
| 4 O | 117.440 | 151.459 | 141.405 | 4 C | 120.830 | 149.754 | 148.979 |
| 5 C | 119.990 | 150.808 | 139.438 | 5 C | 120.195 | 149.948 | 146.656 |
| 6 C | 120.865 | 151.993 | 139.143 | 6 C | 119.967 | 152.942 | 138.112 |
| 7 C | 122.213 | 151.968 | 139.453 | 7 C | 121.630 | 151.197 | 138.273 |
| 8 C | 120.349 | 153.136 | 138.558 | 8 C | 120.923 | 153.888 | 138.480 |
| 9 C | 123.026 | 153.050 | 139.191 | 9 C | 122.586 | 152.143 | 138.642 |
| 10 C | 121.157 | 154.227 | 138.289 | 10 C | 120.511 | 150.546 | 147.876 |
| 11 C | 122.494 | 154.175 | 138.606 | 11 C | 120.320 | 151.597 | 138.008 |
| 12 O | 123.302 | 155.254 | 138.342 | 12 C | 122.233 | 153.489 | 138.745 |
| 13 C | 118.125 | 154.001 | 143.534 | 13 C | 118.152 | 152.613 | 146.121 |
| 14 N | 119.116 | 152.928 | 141.617 | 14 C | 117.832 | 151.766 | 142.968 |
| 15 O | 118.538 | 154.955 | 144.185 | 15 C | 119.189 | 154.127 | 148.502 |
| 16 C | 118.226 | 154.002 | 142.029 | 16 C | 117.713 | 150.944 | 139.593 |
| 17 C | 118.737 | 155.338 | 141.536 | 17 C | 118.696 | 153.686 | 141.606 |
| 18 N | 117.595 | 152.911 | 144.082 | 18 C | 116.966 | 151.510 | 148.034 |
| 19 C | 117.551 | 152.699 | 145.517 | 19 C | 120.506 | 152.050 | 148.002 |
| 20 C | 118.944 | 152.630 | 146.119 | 20 C | 119.288 | 150.576 | 137.611 |
| 21 O | 119.893 | 152.361 | 145.381 | 21 C | 116.977 | 152.172 | 145.266 |
| 22 C | 120.881 | 154.394 | 147.485 | 22 C | 120.243 | 155.963 | 149.819 |
| 23 N | 119.047 | 152.847 | 147.437 | 23 C | 121.378 | 156.524 | 148.982 |
| 24 O | 121.664 | 154.429 | 146.538 | 24 C | 117.878 | 152.404 | 141.586 |
| 25 C | 117.939 | 152.991 | 148.347 | 25 C | 119.140 | 152.605 | 148.421 |
| 26 C | 120.305 | 153.144 | 148.128 | 26 C | 118.510 | 149.942 | 138.773 |
| 27 C | 121.394 | 152.051 | 148.125 | 27 N | 117.186 | 152.573 | 143.895 |
| 28 C | 120.910 | 150.669 | 147.924 | 28 N | 120.048 | 154.567 | 149.499 |
| 29 C | 120.042 | 148.064 | 147.544 | 29 N | 118.435 | 151.440 | 140.668 |
| 30 C | 120.038 | 150.085 | 148.843 | 30 N | 118.089 | 152.257 | 147.473 |
| 31 C | 121.353 | 149.941 | 146.821 | 31 N | 117.617 | 148.862 | 138.262 |
| 32 C | 119.598 | 148.786 | 148.646 | 32 O | 119.091 | 153.243 | 145.627 |
| 33 C | 120.918 | 148.637 | 146.627 | 33 O | 118.286 | 150.649 | 143.207 |
| 34 N | 120.253 | 155.512 | 148.116 | 34 O | 118.505 | 154.857 | 147.788 |
| 35 O | 119.037 | 158.187 | 147.081 | 35 O | 116.559 | 151.264 | 139.316 |
| 36 C | 120.891 | 156.702 | 147.563 | 36 O | 123.167 | 154.412 | 139.103 |
| 37 C | 120.060 | 157.367 | 146.442 | 37 O | 122.579 | 155.827 | 149.287 |

Table S16. Atom matching between DAMGO atoms in the experimental pose and the re-docked pose.

| Experimental pose | | | | Re-docked pose | | | |
|---|---|---|---|---|---|---|---|
| Atom index | Coordinates (x, y, z) | | | Atom index | Coordinates (x, y, z) | | |
| 1 N | 119.032 | 149.353 | 141.168 | 31 N | 117.617 | 148.862 | 138.262 |
| 2 C | 119.627 | 150.658 | 140.911 | 26 C | 118.510 | 149.942 | 138.773 |
| 3 C | 118.634 | 151.725 | 141.330 | 16 C | 117.713 | 150.944 | 139.593 |
| 4 O | 117.440 | 151.459 | 141.405 | 35 O | 116.559 | 151.264 | 139.316 |
| 5 C | 119.990 | 150.808 | 139.438 | 20 C | 119.288 | 150.576 | 137.611 |
| 6 C | 120.865 | 151.993 | 139.143 | 11 C | 120.320 | 151.597 | 138.008 |
| 7 C | 122.213 | 151.968 | 139.453 | 7 C | 121.630 | 151.197 | 138.273 |
| 8 C | 120.349 | 153.136 | 138.558 | 6 C | 119.967 | 152.942 | 138.112 |
| 9 C | 123.026 | 153.050 | 139.191 | 9 C | 122.586 | 152.143 | 138.642 |
| 10 C | 121.157 | 154.227 | 138.289 | 8 C | 120.923 | 153.888 | 138.480 |
| 11 C | 122.494 | 154.175 | 138.606 | 12 C | 122.233 | 153.489 | 138.745 |
| 12 O | 123.302 | 155.254 | 138.342 | 36 O | 123.167 | 154.412 | 139.103 |
| 13 C | 118.125 | 154.001 | 143.534 | 14 C | 117.832 | 151.766 | 142.968 |
| 14 N | 119.116 | 152.928 | 141.617 | 29 N | 118.435 | 151.440 | 140.668 |
| 15 O | 118.538 | 154.955 | 144.185 | 33 O | 118.286 | 150.649 | 143.207 |
| 16 C | 118.226 | 154.002 | 142.029 | 24 C | 117.878 | 152.404 | 141.586 |
| 17 C | 118.737 | 155.338 | 141.536 | 17 C | 118.696 | 153.686 | 141.606 |
| 18 N | 117.595 | 152.911 | 144.082 | 27 N | 117.186 | 152.573 | 143.895 |
| 19 C | 117.551 | 152.699 | 145.517 | 21 C | 116.977 | 152.172 | 145.266 |
| 20 C | 118.944 | 152.630 | 146.119 | 13 C | 118.152 | 152.613 | 146.121 |
| 21 O | 119.893 | 152.361 | 145.381 | 32 O | 119.091 | 153.243 | 145.627 |
| 22 C | 120.881 | 154.394 | 147.485 | 15 C | 119.189 | 154.127 | 148.502 |
| 23 N | 119.047 | 152.847 | 147.437 | 30 N | 118.089 | 152.257 | 147.473 |
| 24 O | 121.664 | 154.429 | 146.538 | 34 O | 118.505 | 154.857 | 147.788 |
| 25 C | 117.939 | 152.991 | 148.347 | 18 C | 116.966 | 151.510 | 148.034 |
| 26 C | 120.305 | 153.144 | 148.128 | 25 C | 119.140 | 152.605 | 148.421 |
| 27 C | 121.394 | 152.051 | 148.125 | 19 C | 120.506 | 152.050 | 148.002 |
| 28 C | 120.910 | 150.669 | 147.924 | 10 C | 120.511 | 150.546 | 147.876 |
| 29 C | 120.042 | 148.064 | 147.544 | 1 C | 120.519 | 147.766 | 147.642 |
| 30 C | 120.038 | 150.085 | 148.843 | 4 C | 120.830 | 149.754 | 148.979 |
| 31 C | 121.353 | 149.941 | 146.821 | 5 C | 120.195 | 149.948 | 146.656 |
| 32 C | 119.598 | 148.786 | 148.646 | 2 C | 120.834 | 148.364 | 148.862 |
| 33 C | 120.918 | 148.637 | 146.627 | 3 C | 120.199 | 148.558 | 146.539 |
| 34 N | 120.253 | 155.512 | 148.116 | 28 N | 120.048 | 154.567 | 149.499 |
| 35 O | 119.037 | 158.187 | 147.081 | 37 O | 122.579 | 155.827 | 149.287 |
| 36 C | 120.891 | 156.702 | 147.563 | 22 C | 120.243 | 155.963 | 149.819 |
| 37 C | 120.060 | 157.367 | 146.442 | 23 C | 121.378 | 156.524 | 148.982 |

Table S17. RMSD values for the re-docked poses of DAMGO with respect to the experimental pose calculated by using ProRMSD, DockRMSD, and LigRMSD.

| | ProRMSD | DockRMSD | LigRMSD |
|---|---|---|---|
| **RMSD** | 1.973 | 1.973 | 1.97 |

Note: RMSD value for the web server LigRMSD was reported with two decimal digits, as this is how the value is reported by the web server.

Figure S7. Analysis of re-docking results of the structure of the μ-opioid receptor (μOR)-$G_i$ protein complex bound to the agonist peptide DAMGO. Experimental pose of the ligand in ball and stick representation and with carbon, nitrogen, and oxygen atoms coloured by element: grey, blue, and red, respectively. Docked pose of the ligand in licorice representation and with carbon, nitrogen, oxygen, and hydrogen atoms coloured in light blue, blue, red, and white, respectively. Receptor in cartoon representation, with atom index colour style. (A) Atom indexes of the experimental pose of the ligand. (B) Atom indexes of the docked pose of the ligand assigned by OpenEye HYBRID docking program. (C) Ligand docked pose compared with ligand experimental pose. (D) Ligand docked pose within the receptor binding pocket. (E) Interactions between the receptor and the ligand in the docked pose within the binding pocket. Selected residues from the receptor are in licorice representation with carbon, oxygen, nitrogen, and sulphur atoms coloured light grey, red, blue, and yellow, respectively. These residues are labelled in black and selected interactions are shown as dashed lines. The ligand atoms involved in the main interactions are surrounded by a green sphere. The receptor cartoon representation has been opacified for clarity.

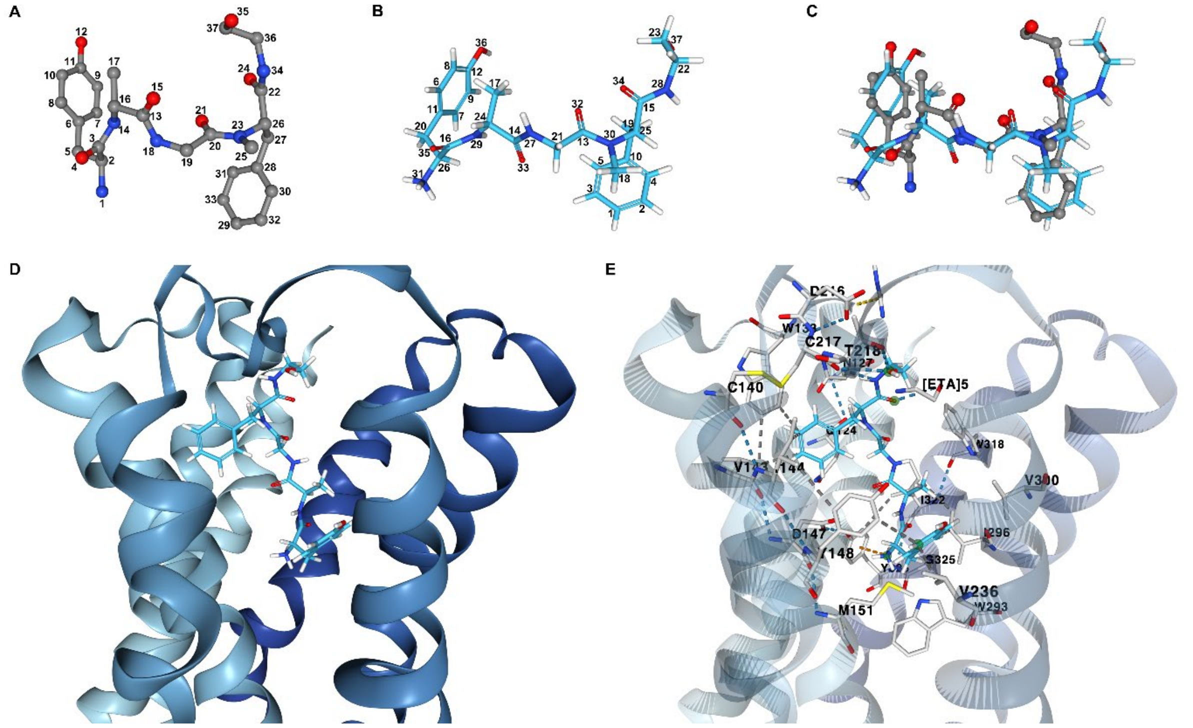

ClusDOCK performs the cluster analysis with three different algorithms. In this example, the gromos algorithm was employed, with a cut-off of 1.0 Å and a minimum cluster size of 1 structure. The steps that lead to cluster formations are illustrated below, in Table S18-S19.

Table S18. Pairwise RMSD matrix (20×20) in which each element contains the RMSD of the i$^{th}$ binding pose calculated with respect to the j$^{th}$ binding pose of DAMGO re-docking. Considering a cut-off of 1.0 Å, the number of neighbours is calculated (RMSD values below the cut-off are highlighted in gold). There are multiple structures that have the highest number of neighbours, i.e., ten. Among these, the first pose (highlighted in green) has the best scoring function, thus it represents the centre of the most populated cluster, and forms together with all its neighbours (poses no. 2, 3, 4, 5, 7, 8, 9, 10, 13, 17, and 18; highlighted in yellow) a cluster. Therefore, the first cluster is composed of structures 1, 2, 3, 4, 5, 7, 8, 9, 10, 13, 17, and 18; and presents the scoring function of structure 1, the centroid of the cluster. These structures are thereafter eliminated from the pool of structures.

| Pose no. | 1 | 2 | 3 | 4 | 5 | 6 | 7 | 8 | 9 | 10 | 11 | 12 | 13 | 14 | 15 | 16 | 17 | 18 | 19 | 20 |
|---|---|---|---|---|---|---|---|---|---|---|---|---|---|---|---|---|---|---|---|---|
| 1 | 0 | 0.196 | 0.588 | 0.521 | 0.613 | 9.166 | 0.413 | 0.564 | 0.466 | 0.270 | 9.126 | 9.180 | 0.769 | 9.117 | 9.180 | 9.186 | 0.668 | 0.641 | 9.104 | 9.106 |
| 2 | 0.196 | 0 | 0.465 | 0.455 | 0.536 | 9.152 | 0.375 | 0.488 | 0.408 | 0.174 | 9.115 | 9.166 | 0.700 | 9.108 | 9.156 | 9.172 | 0.557 | 0.536 | 9.095 | 9.096 |
| 3 | 0.588 | 0.465 | 0 | 0.432 | 0.297 | 9.083 | 0.327 | 0.380 | 0.519 | 0.531 | 9.055 | 9.098 | 0.409 | 9.055 | 9.089 | 9.105 | 0.397 | 0.480 | 9.042 | 9.034 |
| 4 | 0.521 | 0.455 | 0.432 | 0 | 0.607 | 9.148 | 0.497 | 0.593 | 0.174 | 0.558 | 9.117 | 9.165 | 0.707 | 9.117 | 9.155 | 9.169 | 0.431 | 0.421 | 9.099 | 9.090 |
| 5 | 0.613 | 0.536 | 0.297 | 0.607 | 0 | 9.075 | 0.295 | 0.244 | 0.707 | 0.601 | 9.044 | 9.091 | 0.174 | 9.041 | 9.061 | 9.099 | 0.515 | 0.630 | 9.030 | 9.024 |
| 6 | 9.166 | 9.152 | 9.083 | 9.148 | 9.075 | 0 | 9.092 | 9.090 | 9.166 | 9.143 | 0.309 | 0.157 | 9.066 | 0.500 | 1.478 | 0.318 | 9.224 | 9.255 | 0.379 | 0.425 |
| 7 | 0.413 | 0.375 | 0.327 | 0.497 | 0.295 | 9.092 | 0 | 0.237 | 0.552 | 0.399 | 9.058 | 9.105 | 0.438 | 9.050 | 9.093 | 9.112 | 0.496 | 0.595 | 9.042 | 9.043 |
| 8 | 0.564 | 0.488 | 0.380 | 0.593 | 0.244 | 9.090 | 0.237 | 0 | 0.677 | 0.512 | 9.057 | 9.104 | 0.338 | 9.050 | 9.058 | 9.111 | 0.476 | 0.616 | 9.041 | 9.042 |
| 9 | 0.466 | 0.408 | 0.519 | 0.174 | 0.707 | 9.166 | 0.552 | 0.677 | 0 | 0.492 | 9.133 | 9.182 | 0.832 | 9.131 | 9.183 | 9.185 | 0.527 | 0.481 | 9.114 | 9.108 |
| 10 | 0.270 | 0.174 | 0.531 | 0.558 | 0.601 | 9.143 | 0.399 | 0.512 | 0.492 | 0 | 9.105 | 9.154 | 0.769 | 9.094 | 9.148 | 9.160 | 0.627 | 0.638 | 9.086 | 9.093 |
| 11 | 9.126 | 9.115 | 9.055 | 9.117 | 9.044 | 0.309 | 9.058 | 9.057 | 9.133 | 9.105 | 0 | 0.326 | 9.037 | 0.243 | 1.483 | 0.448 | 9.195 | 9.226 | 0.308 | 0.478 |
| 12 | 9.180 | 9.166 | 9.098 | 9.165 | 9.091 | 0.157 | 9.105 | 9.104 | 9.182 | 9.154 | 0.326 | 0 | 9.083 | 0.453 | 1.489 | 0.287 | 9.240 | 9.272 | 0.433 | 0.567 |
| 13 | 0.769 | 0.700 | 0.409 | 0.707 | 0.174 | 9.066 | 0.438 | 0.338 | 0.832 | 0.769 | 9.037 | 9.083 | 0 | 9.036 | 9.041 | 9.092 | 0.579 | 0.710 | 9.023 | 9.014 |
| 14 | 9.117 | 9.108 | 9.055 | 9.117 | 9.041 | 0.500 | 9.050 | 9.050 | 9.131 | 9.094 | 0.243 | 0.453 | 9.036 | 0 | 1.547 | 0.563 | 9.195 | 9.228 | 0.412 | 0.679 |
| 15 | 9.180 | 9.156 | 9.089 | 9.155 | 9.061 | 1.478 | 9.093 | 9.058 | 9.183 | 9.148 | 1.483 | 1.489 | 9.041 | 1.547 | 0 | 1.514 | 9.203 | 9.246 | 1.414 | 1.495 |
| 16 | 9.186 | 9.172 | 9.105 | 9.169 | 9.099 | 0.318 | 9.112 | 9.111 | 9.185 | 9.160 | 0.448 | 0.287 | 9.092 | 0.563 | 1.514 | 0 | 9.247 | 9.280 | 0.486 | 0.608 |
| 17 | 0.668 | 0.557 | 0.397 | 0.431 | 0.515 | 9.224 | 0.496 | 0.476 | 0.527 | 0.627 | 9.195 | 9.240 | 0.579 | 9.195 | 9.203 | 9.247 | 0 | 0.230 | 9.179 | 9.172 |
| 18 | 0.641 | 0.536 | 0.480 | 0.421 | 0.630 | 9.255 | 0.595 | 0.616 | 0.481 | 0.638 | 9.226 | 9.272 | 0.710 | 9.228 | 9.246 | 9.280 | 0.230 | 0 | 9.207 | 9.196 |
| 19 | 9.104 | 9.095 | 9.042 | 9.099 | 9.030 | 0.379 | 9.042 | 9.041 | 9.114 | 9.086 | 0.308 | 0.433 | 9.023 | 0.412 | 1.414 | 0.486 | 9.179 | 9.207 | 0 | 0.357 |
| 20 | 9.106 | 9.096 | 9.034 | 9.090 | 9.024 | 0.425 | 9.043 | 9.042 | 9.108 | 9.093 | 0.478 | 0.567 | 9.014 | 0.679 | 1.495 | 0.608 | 9.172 | 9.196 | 0.357 | 0 |
| No. of neighbours | 11 | 11 | 11 | 11 | 11 | 6 | 11 | 11 | 11 | 11 | 6 | 6 | 11 | 6 | 0 | 6 | 11 | 11 | 6 | 6 |

Table S19. Pairwise RMSD matrix (8×8) recalculated. Among the structures that have the highest number of neighbours, the number 6 has the best scoring function, thus it represents the centre of the second cluster, which includes its neighbour (poses no. 11, 12, 14, 16, 19, and 20), and presents the scoring function of structure 6. These structures are thereafter eliminated from the pool of structures.

| Pose no. | 6 | 11 | 12 | 14 | 15 | 16 | 19 | 20 |
|---|---|---|---|---|---|---|---|---|
| 6 | 0 | 0.309 | 0.157 | 0.500 | 1.478 | 0.318 | 0.379 | 0.425 |
| 11 | 0.309 | 0 | 0.326 | 0.243 | 1.483 | 0.448 | 0.308 | 0.478 |
| 12 | 0.157 | 0.326 | 0 | 0.453 | 1.489 | 0.287 | 0.433 | 0.567 |
| 14 | 0.500 | 0.243 | 0.453 | 0 | 1.547 | 0.563 | 0.412 | 0.679 |
| 15 | 1.478 | 1.483 | 1.489 | 1.547 | 0 | 1.514 | 1.414 | 1.495 |
| 16 | 0.318 | 0.448 | 0.287 | 0.563 | 1.514 | 0 | 0.486 | 0.608 |
| 19 | 0.379 | 0.308 | 0.433 | 0.412 | 1.414 | 0.486 | 0 | 0.357 |
| 20 | 0.425 | 0.478 | 0.567 | 0.679 | 1.495 | 0.608 | 0.357 | 0 |
| No. of neighbours | 6 | 6 | 6 | 6 | 0 | 6 | 6 | 6 |

Structure number 15 remains as a singleton cluster with its scoring function.

Figure S8. Result of cluster analysis with the gromos algorithm on DAMGO re-docking results. (A) For each cluster is reported the cluster population, the structure indexes, and the scoring function of the centroid of the cluster that was selected as the most representative structure. The order of the clusters depends on their scoring function (the first one has the lowest scoring function). (B) Bar plot that shows on the y-axis the population for each cluster and on the x-axis the scoring function of the centroid of each cluster. The representative structure of each cluster is also reported.

| CLUSTER | #CONFORMATIONS | STRUCTURES | SCORING FUNCTION |
|---|---|---|---|
| 1 | 12 | 1, 2, 3, 4, 5, 7, 8, 9, 10, 13, 17, 18 | -9.813098 |
| 2 | 7 | 6, 11, 12, 14, 16, 19, 20 | -9.395459 |
| 3 | 1 | 15 | -8.892843 |

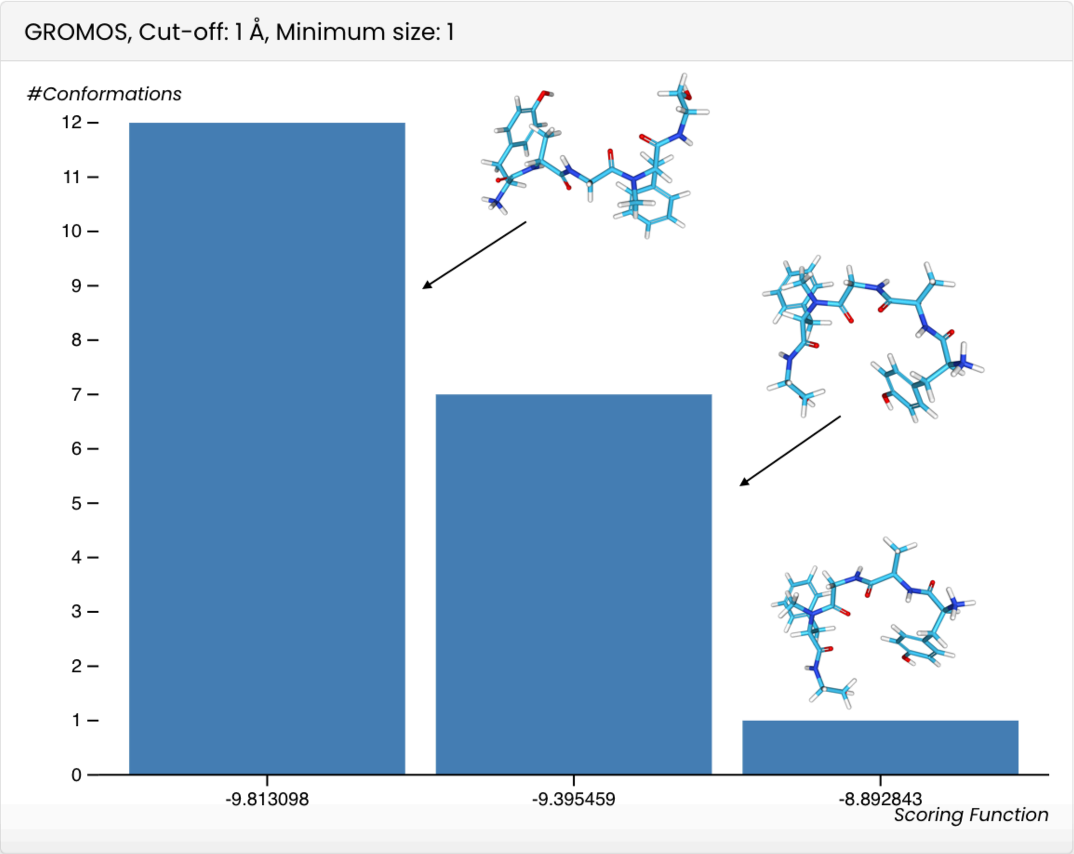

Figure S9. Result of cluster analysis with the single-linkage algorithm on DAMGO re-docking results. (A) For each cluster is reported the cluster population, the structure indexes, and the scoring function of the centroid of the cluster that was selected as the most representative structure. The order of the clusters depends on their scoring function (the first one has the lowest scoring function). (B) Bar plot that shows on the y-axis the population for each cluster and on the x-axis the scoring function of the centroid of each cluster. The representative structure of each cluster is also reported.

| CLUSTER | #CONFORMATIONS | STRUCTURES | SCORING FUNCTION |
|---|---|---|---|
| 1 | 12 | 1, 2, 3, 4, 5, 7, 8, 9, 10, 13, 17, 18 | -9.813098 |
| 2 | 7 | 6, 11, 12, 14, 16, 19, 20 | -9.395459 |
| 3 | 1 | 15 | -8.892843 |

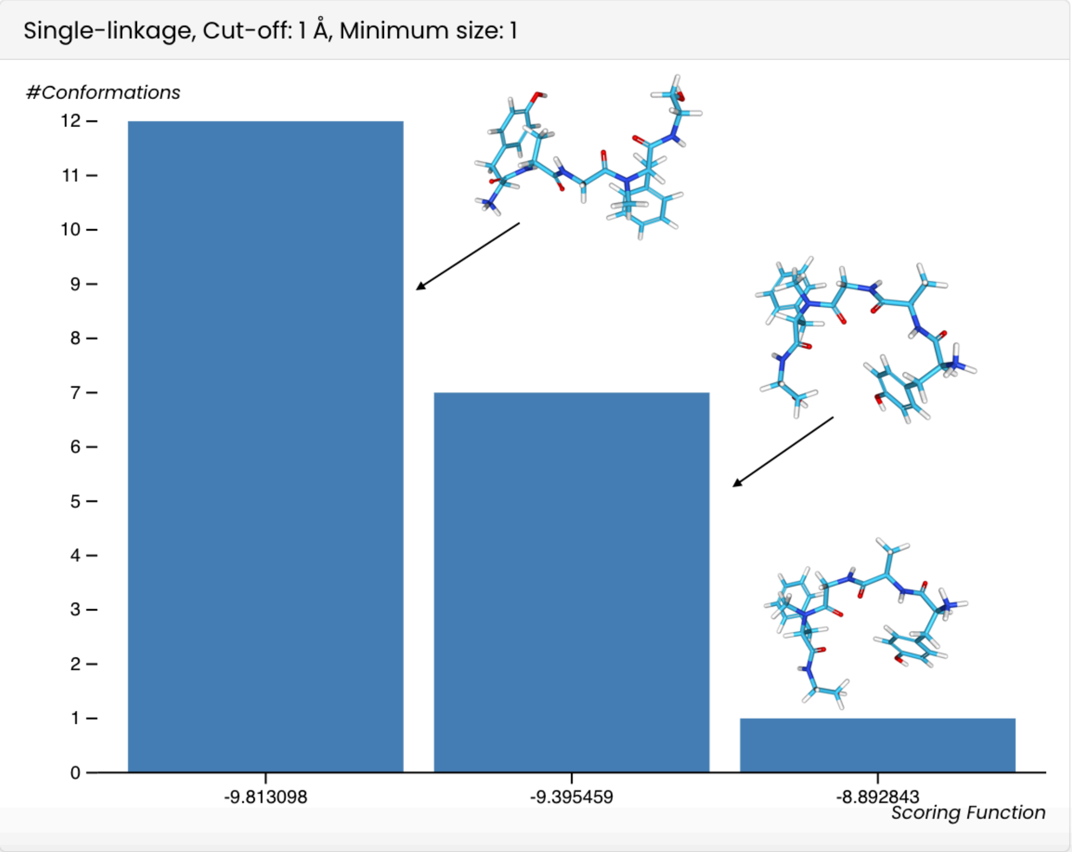

Figure S10. Result of cluster analysis with the complete-linkage algorithm on DAMGO re-docking results. (A) For each cluster is reported the cluster population, the structure indexes, and the scoring function of the centroid of the cluster that was selected as the most representative structure. The order of the clusters depends on their scoring function (the first one has the lowest scoring function). (B) Bar plot that shows on the y-axis the population for each cluster and on the x-axis the scoring function of the centroid of each cluster. The representative structure of each cluster is also reported.

| CLUSTER | #CONFORMATIONS | STRUCTURES | SCORING FUNCTION |
|---|---|---|---|
| 1 | 12 | 1, 2, 3, 4, 5, 7, 8, 9, 10, 13, 17, 18 | -9.813098 |
| 2 | 7 | 6, 11, 12, 14, 16, 19, 20 | -9.395459 |
| 3 | 1 | 15 | -8.892843 |

Complete-linkage, Cut-off: 1 Å, Minimum size: 1

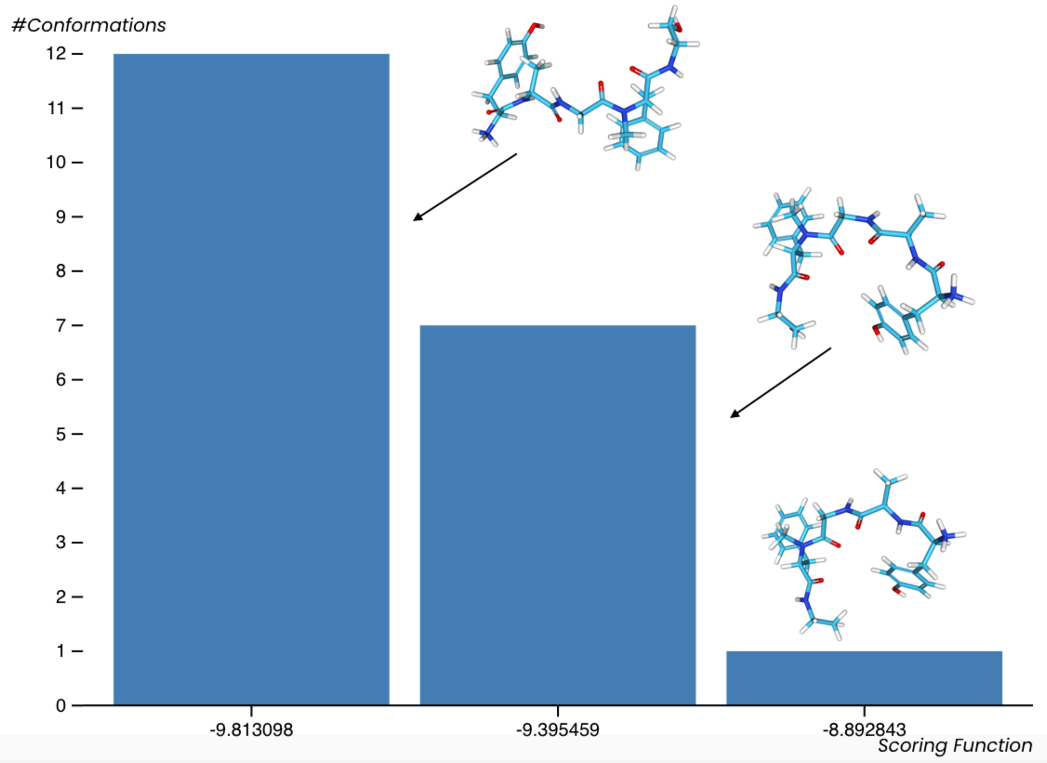


In this example, the three clustering algorithms are able to distinguish between different binding poses and give the same results.

## S5 COMPARISON WITH SIMILAR WEB SERVERS

Table S20. Comparison of functionalities between different web servers for RMSD calculations.

| Web Server | Input file formats | Reference pose vs docked pose | Reference pose vs multiple docked poses | RMSD among similar compounds | Superimposed RMSD | Backbone RMSD |
|---|---|---|---|---|---|---|
| PacDOCK (ProRMSD) | SDF, PDB, MOL2, MOL | YES | YES | NO | YES | YES |
| DockRMSD | MOL2 | YES | NO | NO | NO | NO |
| LigRMSD | MOL | YES | YES* | YES | NO | NO |

*Each docked pose must be contained in its own file and a zip file of all MOL files must be uploaded.

To test ProRMSD ability and to compare its performance with DockRMSD and LigRMSD, the ligands of ten different target-ligand complex structures downloaded from RCSB PDB were separated from the target, and then re-docked into the target using the docking program OpenEye HYBRID (*see Section S1).*

The RMSD values of the first tenth docked poses with respect to the reference experimental pose were calculated with the three different web servers.

Note: RMSD values for the web server LigRMSD were reported with two decimal digits, as this is how the value is reported by the web server.

Table S21. List of PDB identification codes of the selected complex structures for re-docking.

| PDB ID | CLASSIFICATION | LIGAND | METHOD | RESOLUTION (Å) |
|---|---|---|---|---|
| 1DNE | DNA | NT | X-ray | 2.40 |
| 2PIV | Hormone receptor | T3 | X-ray | 1.95 |
| 3LFA | Transferase | 1N1 | X-ray | 2.10 |
| 4EIY | Membrane protein | ZMA | X-ray | 1.80 |
| 4LRM | Transferase/Transferase inhibitor | YUN | X-ray | 3.53 |
| 5CGC | Signaling protein | 51D | X-ray | 3.10 |
| 6DDF | Membrane Protein | DAMGO | cryo-EM | 3.50 |
| 6DUK | Transferase/Transferase inhibitor | JBJ | X-ray | 2.20 |
| 6ZKK | Electron transport | 970 | cryo-EM | 3.70 |
| 7LGS | Hydrolase/Hydrolase inhibitor | Q6K | X-ray | 3.10 |

The complex structures used for this test were chosen on the basis of their ligands, in an attempt to represent different classes of molecules of pharmaceutical interest, with criteria outlined in Table S22.

Table S22. Re-docking structures selection criteria.

| CRITERIA | LIGAND |
|---|---|
| PARTIAL SYMMETRY | NT |
| HIGHLY FLEXIBILITY | 1N1; DAMGO |
| HETEROATOMS | T3; JBJ; 51D |
| MULTIPLE RINGS | Q6K; ZMA |
| CONDENSED RINGS | 970; YUN |

Table S23. RMSD values of NT re-docking.

| 1DNE - NT | ProRMSD | DockRMSD | Absolute difference (Å) | Relative difference (%) | LigRMSD | Absolute difference (Å) | Relative difference (%) |
|---|---|---|---|---|---|---|---|
| **POSE 1** | 2.219 | 2.219 | | | 2.22 | | |
| **POSE 2** | 2.100 | 2.100 | | | 2.10 | | |
| **POSE 3** | 11.796 | 11.796 | | | 11.78 | 0.02 | 0.14 |
| **POSE 4** | 4.053 | 4.053 | | | 4.05 | | |
| **POSE 5** | 11.759 | 11.759 | | | 11.74 | 0.02 | 0.16 |
| **POSE 6** | 11.819 | 11.818 | | | 11.81 | 0.01 | 0.08 |
| **POSE 7** | 4.382 | 4.382 | | | 4.35 | 0.03 | 0.74 |
| **POSE 8** | 11.789 | 11.789 | | | 11.77 | 0.02 | 0.16 |
| **POSE 9** | 4.167 | 4.167 | | | 4.17 | | |
| **POSE 10** | 2.068 | 2.068 | | | 2.07 | | |

Figure S11. NT ligand.

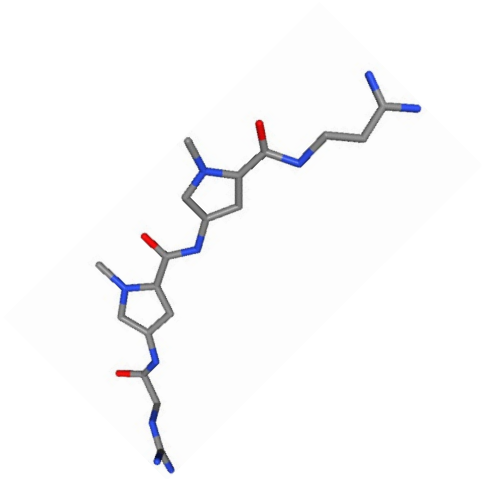

Table S24. RMSD values of T3 re-docking.

| 2PIV - T3 | ProRMSD | DockRMSD | Absolute difference (Å) | Relative difference (%) | LigRMSD | Absolute difference (Å) | Relative difference (%) |
|---|---|---|---|---|---|---|---|
| **POSE 1** | 2.999 | 2.999 | | | 2.63 | 0.37 | 14.03 |
| **POSE 2** | 2.985 | 2.985 | | | 2.62 | 0.37 | 13.93 |
| **POSE 3** | 2.852 | 2.852 | | | 2.43 | 0.42 | 17.37 |
| **POSE 4** | 2.874 | 2.874 | | | 2.45 | 0.42 | 17.31 |
| **POSE 5** | 2.934 | 2.934 | | | 2.56 | 0.37 | 14.61 |
| **POSE 6** | 2.931 | 2.931 | | | 2.48 | 0.45 | 18.19 |
| **POSE 7** | 2.868 | 2.868 | | | 2.37 | 0.50 | 21.01 |
| **POSE 8** | 3.090 | 3.09 | | | 2.77 | 0.32 | 11.55 |
| **POSE 9** | 2.946 | 2.946 | | | 2.58 | 0.37 | 14.19 |
| **POSE 10** | 3.166 | 3.166 | | | 2.65 | 0.52 | 19.47 |

Figure S12. T3 ligand.

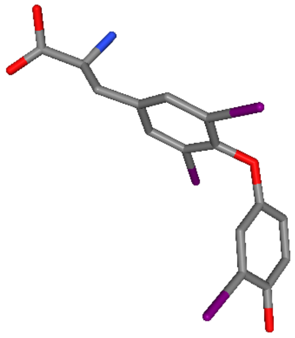

Table S25. RMSD values of 1N1 re-docking.

| 3LFA – 1N1 | ProRMSD | DockRMSD | Absolute difference (Å) | Relative difference (%) | LigRMSD | Absolute difference (Å) | Relative difference (%) |
|---|---|---|---|---|---|---|---|
| **POSE 1** | 11.857 | 11.847 | 0.010 | 0.08 | 11.85 | 0.01 | 0.06 |
| **POSE 2** | 11.857 | 11.850 | 0.007 | 0.06 | 11.85 | 0.01 | 0.06 |
| **POSE 3** | 11.853 | 11.845 | 0.008 | 0.07 | 11.85 | | |
| **POSE 4** | 11.854 | 11.844 | 0.010 | 0.08 | 11.84 | 0.01 | 0.12 |
| **POSE 5** | 11.854 | 11.844 | 0.010 | 0.08 | 11.84 | 0.01 | 0.12 |
| **POSE 6** | 11.860 | 11.853 | 0.007 | 0.06 | 11.85 | 0.01 | 0.08 |
| **POSE 7** | 11.845 | 11.837 | 0.008 | 0.07 | 11.84 | 0.01 | 0.04 |
| **POSE 8** | 11.768 | 11.760 | 0.008 | 0.07 | 11.76 | 0.01 | 0.07 |
| **POSE 9** | 2.281 | 2.253 | 0.028 | 1.24 | 2.25 | 0.03 | 1.38 |
| **POSE 10** | 11.848 | 11.840 | 0.008 | 0.07 | 11.84 | 0.01 | 0.07 |

Figure S13. 1N1 ligand.

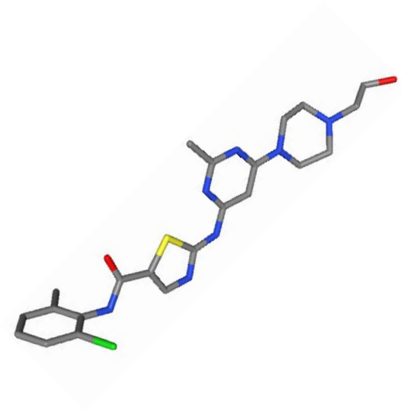

Table S26. RMSD values of ZMA re-docking.

| 4EIY - ZMA | ProRMSD | DockRMSD | Absolute difference (Å) | Relative difference (%) | LigRMSD | Absolute difference (Å) | Relative difference (%) |
|---|---|---|---|---|---|---|---|
| **POSE 1** | 1.431 | 1.431 | | | 1.07 | 0.36 | 33.74 |
| **POSE 2** | 1.570 | 1.570 | | | 1.25 | 0.32 | 25.60 |
| **POSE 3** | 1.449 | 1.449 | | | 1.09 | 0.36 | 32.94 |
| **POSE 4** | 1.532 | 1.532 | | | 1.20 | 0.33 | 27.67 |
| **POSE 5** | 1.487 | 1.487 | | | 1.14 | 0.35 | 30.44 |
| **POSE 6** | 1.644 | 1.644 | | | 1.34 | 0.30 | 22.69 |
| **POSE 7** | 1.661 | 1.661 | | | 1.36 | 0.30 | 22.13 |
| **POSE 8** | 2.205 | 2.205 | | | 2.21 | | |
| **POSE 9** | 2.219 | 2.219 | | | 2.22 | | |
| **POSE 10** | 2.317 | 2.317 | | | 2.32 | | |

Figure S14. ZMA ligand.

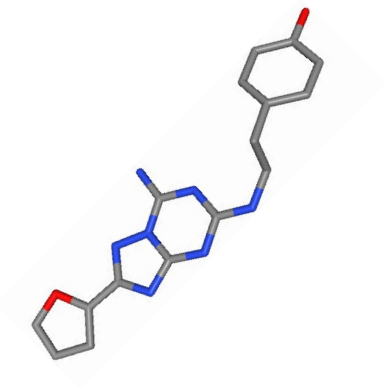

Table S27. RMSD values of YUN re-docking.

| 4LRM - YUN | ProRMSD | DockRMSD | Absolute difference (Å) | Relative difference (%) | LigRMSD | Absolute difference (Å) | Relative difference (%) |
|---|---|---|---|---|---|---|---|
| **POSE 1** | 6.410 | 6.410 | | | 6.41 | | |
| **POSE 2** | 6.413 | 6.413 | | | 6.41 | | |
| **POSE 3** | 6.715 | 6.715 | | | 6.71 | | |
| **POSE 4** | 6.589 | 6.589 | | | 6.59 | | |
| **POSE 5** | 6.597 | 6.597 | | | 6.60 | | |
| **POSE 6** | 6.514 | 6.514 | | | 6.51 | | |
| **POSE 7** | 6.559 | 6.559 | | | 6.56 | | |
| **POSE 8** | 6.570 | 6.570 | | | 6.57 | | |
| **POSE 9** | 6.530 | 6.530 | | | 6.53 | | |
| **POSE 10** | 6.520 | 6.520 | | | 6.52 | | |

Figure S15. YUN ligand.

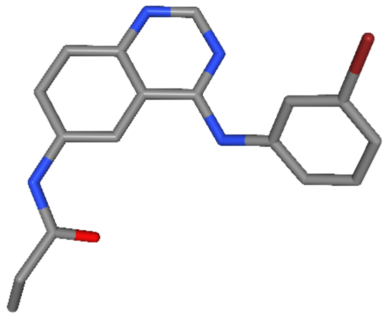

Table S28. RMSD values of 51D re-docking.

| 5CGC – 51D | ProRMSD | DockRMSD | Absolute difference (Å) | Relative difference (%) | LigRMSD | Absolute difference (Å) | Relative difference (%) |
|---|---|---|---|---|---|---|---|
| **POSE 1** | 0.297 | 0.297 | | | 0.30 | | |
| **POSE 2** | 0.427 | 0.427 | | | 0.43 | | |
| **POSE 3** | 0.293 | 0.293 | | | 0.29 | | |
| **POSE 4** | 0.264 | 0.264 | | | 0.26 | | |
| **POSE 5** | 0.535 | 0.535 | | | 0.53 | | |
| **POSE 6** | 0.538 | 0.538 | | | 0.54 | | |
| **POSE 7** | 2.681 | 2.681 | | | 2.68 | | |
| **POSE 8** | 0.395 | 0.395 | | | 0.39 | | |
| **POSE 9** | 0.411 | 0.411 | | | 0.41 | | |
| **POSE 10** | 0.906 | 0.906 | | | 0.91 | | |

Figure S16. 51D ligand.

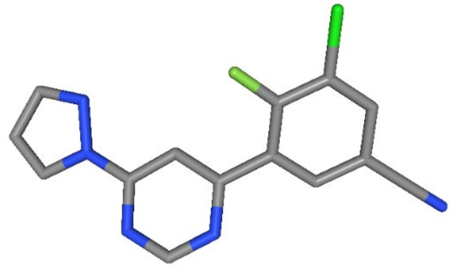

Table S29. RMSD values of DAMGO re-docking.

| 6DDF - DAMGO | ProRMSD | DockRMSD | Absolute difference (Å) | Relative difference (%) | LigRMSD | Absolute difference (Å) | Relative difference (%) |
|---|---|---|---|---|---|---|---|
| **POSE 1** | 1.973 | 1.973 | | | 1.97 | | |
| **POSE 2** | 1.974 | 1.974 | | | 1.97 | | |
| **POSE 3** | 1.910 | 1.910 | | | 1.91 | | |
| **POSE 4** | 1.999 | 1.999 | | | 2.00 | | |
| **POSE 5** | 1.864 | 1.864 | | | 1.86 | | |
| **POSE 6** | 8.400 | 8.400 | | | 8.38 | 0.02 | 0.24 |
| **POSE 7** | 1.843 | 1.843 | | | 1.84 | | |
| **POSE 8** | 1.848 | 1.848 | | | 1.85 | | |
| **POSE 9** | 2.034 | 2.034 | | | 2.03 | | |
| **POSE 10** | 1.956 | 1.956 | | | 1.96 | | |

Figure S17. DAMGO ligand.

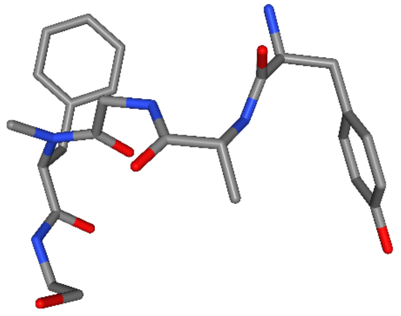

Table S30. RMSD values of JBJ re-docking.

| 6DUK - JBJ | ProRMSD | DockRMSD | Absolute difference (Å) | Relative difference (%) | LigRMSD | Absolute difference (Å) | Relative difference (%) |
|---|---|---|---|---|---|---|---|
| **POSE 1** | 1.188 | 1.188 | | | 1.01 | 0.18 | 17.62 |
| **POSE 2** | 1.228 | 1.228 | | | 1.02 | 0.21 | 20.39 |
| **POSE 3** | 1.217 | 1.217 | | | 1.01 | 0.21 | 20.50 |
| **POSE 4** | 1.337 | 1.337 | | | 1.15 | 0.19 | 16.26 |
| **POSE 5** | 1.363 | 1.363 | | | 1.18 | 0.18 | 15.51 |
| **POSE 6** | 1.207 | 1.207 | | | 1.00 | 0.21 | 20.70 |
| **POSE 7** | 1.348 | 1.348 | | | 1.19 | 0.16 | 13.28 |
| **POSE 8** | 1.205 | 1.205 | | | 0.93 | 0.28 | 29.57 |
| **POSE 9** | 1.283 | 1.283 | | | 1.05 | 0.23 | 22.19 |
| **POSE 10** | 1.330 | 1.330 | | | 1.15 | 0.18 | 15.65 |

Figure S18. JBJ ligand.

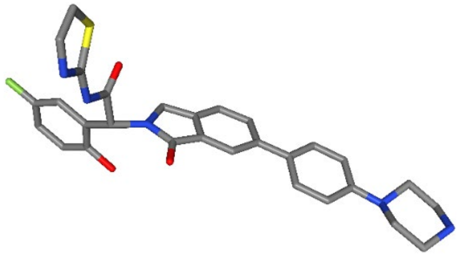

Table S31. RMSD values of 970 re-docking.

| 6ZKK – 970 | ProRMSD | DockRMSD | Absolute difference (Å) | Relative difference (%) | LigRMSD | Absolute difference (Å) | Relative difference (%) |
|---|---|---|---|---|---|---|---|
| POSE 1 | 0.668 | 0.668 | | | 0.67 | | |
| POSE 2 | 8.110 | 8.110 | | | 8.11 | | |
| POSE 3 | 1.217 | 1.217 | | | 1.22 | | |
| POSE 4 | 7.722 | 7.722 | | | 7.72 | | |
| POSE 5 | 7.009 | 7.009 | | | 7.01 | | |
| POSE 6 | 4.536 | 4.536 | | | 4.54 | | |
| POSE 7 | 8.863 | 8.863 | | | 8.86 | | |
| POSE 8 | 7.365 | 7.365 | | | 7.36 | | |
| POSE 9 | 7.850 | 7.850 | | | 7.85 | | |
| POSE 10 | 7.497 | 7.497 | | | 7.50 | | |

Figure S19. 970 ligand.

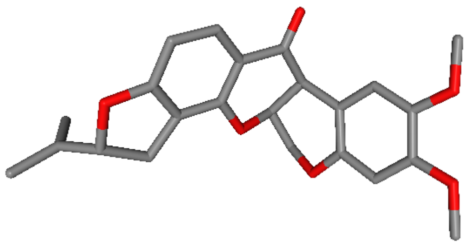

Table S32. RMSD values of Q6K re-docking.

| 7LGS – Q6K | ProRMSD | DockRMSD | Absolute difference (Å) | Relative difference (%) | LigRMSD | Absolute difference (Å) | Relative difference (%) |
|---|---|---|---|---|---|---|---|
| POSE 1 | 1.180 | 1.162 | 0.018 | 1.55 | 1.16 | 0.02 | 1.72 |
| POSE 2 | 1.267 | 1.251 | 0.016 | 1.28 | 1.25 | 0.02 | 1.36 |
| POSE 3 | 2.437 | 2.423 | 0.014 | 0.58 | 2.42 | 0.02 | 0.70 |
| POSE 4 | 1.199 | 1.182 | 0.017 | 1.44 | 1.18 | 0.02 | 1.61 |
| POSE 5 | 2.601 | 2.601 | | | 2.60 | | |
| POSE 6 | 2.302 | 2.285 | 0.017 | 0.74 | 2.28 | 0.02 | 0.96 |
| POSE 7 | 1.321 | 1.306 | 0.015 | 1.15 | 1.31 | 0.01 | 0.84 |
| POSE 8 | 2.612 | 2.612 | | | 2.61 | | |
| POSE 9 | 2.371 | 2.364 | 0.007 | 0.30 | 2.36 | 0.01 | 0.47 |
| POSE 10 | 2.357 | 2.349 | 0.008 | 0.34 | 2.35 | 0.01 | 0.30 |

Figure S20. Q6K ligand .

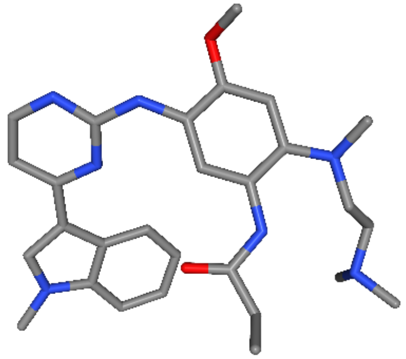

## REFERENCES

1. McGann,M. (2012) FRED and HYBRID docking performance on standardized datasets. *J. Comput. Aided Mol. Des.*, **26**, 897–906.

2. Berman,H.M., Westbrook,J., Feng,Z., Gilliland,G., Bhat,T.N., Weissig,H., Shindyalov,I.N. and Bourne,P.E.. (2000) The Protein Data Bank. *Nucleic Acids Research*, **28**, 235–242.

3. Bell,E.W. and Zhang,Y. (2019) DockRMSD: an open-source tool for atom mapping and RMSD calculation of symmetric molecules through graph isomorphism.

4. Velázquez-Libera,J.L., Durán-Verdugo,F., Valdés-Jiménez,A., Núñez-Vivanco,G. and Caballero,J. (2020) LigRMSD: a web server for automatic structure matching and RMSD calculations among identical and similar compounds in protein-ligand docking. *Bioinformatics*, **36**, 2912–2914.

5. Koehl,A., Hu,H., Maeda,S., Zhang,Y., Qu,Q., Paggi,J.M., Latorraca,N.R., Hilger,D., Dawson,R., Matile,H., *et al.* (2018) Structure of the μ-opioid receptor–Gi protein complex. *Nature*, **558**, 547–552.
